# Dual transcriptomic analysis reveals early induced *Castanea* defense-related genes and *Phytophthora cinnamomi* effectors

**DOI:** 10.3389/fpls.2024.1439380

**Published:** 2024-08-12

**Authors:** Patrícia Fernandes, Diana Pimentel, Ricardo S. Ramiro, Maria do Céu Silva, Pedro Fevereiro, Rita Lourenço Costa

**Affiliations:** ^1^ Department of Environmental Biology, State University of New York College of Environmental Science and Forestry, Syracuse, NY, United States; ^2^ InnovPlantProtect Collaborative Laboratory, Elvas, Portugal; ^3^ Centro de Investigação das Ferrugens do Cafeeiro, Instituto Superior de Agronomia, Universidade de Lisboa, Lisboa, Portugal; ^4^ Linking Landscape, Environment, Agriculture and Food, Associate Laboratory TERRA, Instituto Superior de Agronomia, Universidade de Lisboa, Lisboa, Portugal; ^5^ Instituto de Tecnologia Química e Biológica António Xavier (ITQB, Green-It Unit), Universidade NOVA de Lisboa, Oeiras, Portugal; ^6^ Instituto Nacional de Investigação Agrária e Veterinária I.P., Oeiras, Portugal; ^7^ Centro de Estudos Florestais, Associate Laboratory TERRA, Instituto Superior de Agronomia, Universidade de Lisboa, Lisboa, Portugal

**Keywords:** chestnut, immune response, ink disease, pattern recognition receptors, PAMP, resistance, susceptibility, oomycete

## Abstract

*Phytophthora cinnamomi* Rands devastates forest species worldwide, causing significant ecological and economic impacts. The European chestnut (*Castanea sativa*) is susceptible to this hemibiotrophic oomycete, whereas the Asian chestnuts (*Castanea crenata* and *Castanea mollissima*) are resistant and have been successfully used as resistance donors in breeding programs. The molecular mechanisms underlying the different disease outcomes among chestnut species are a key foundation for developing science-based control strategies. However, these are still poorly understood. Dual RNA sequencing was performed in *C. sativa* and *C. crenata* roots inoculated with *P. cinnamomi.* The studied time points represent the pathogen’s hemibiotrophic lifestyle previously described at the cellular level. *Phytophthora cinnamomi* expressed several genes related to pathogenicity in both chestnut species, such as cell wall–degrading enzymes, host nutrient uptake transporters, and effectors. However, the expression of effectors related to the modulation of host programmed cell death (*elicitins* and *NLPs*) and sporulation-related genes was higher in the susceptible chestnut. After pathogen inoculation, 1,556 and 488 genes were differentially expressed by *C. crenata* and *C. sativa*, respectively. The most significant transcriptional changes occur at 2 h after inoculation (hai) in *C. sativa* and 48 hai in *C. crenata*. Nevertheless, *C. crenata* induced more defense-related genes, indicating that the resistant response to *P. cinnamomi* is controlled by multiple loci, including several pattern recognition receptors, genes involved in the phenylpropanoid, salicylic acid and ethylene/jasmonic acid pathways, and antifungal genes. Importantly, these results validate previously observed cellular responses for *C. crenata*. Collectively, this study provides a comprehensive time-resolved description of the chestnut–*P. cinnamomi* dynamic, revealing new insights into susceptible and resistant host responses and important pathogen strategies involved in disease development.

## 1 Introduction

Plants rely on two layers of their innate immune system to recognize and respond to pathogen infections (Jones and Dangl, 2006). Initially, the host recognizes pathogen-associated molecular patterns (PAMPs) by transmembrane pattern recognition receptors (PRRs) such as receptor-like kinases (RLKs) and receptor-like proteins (RLPs), leading to PTI (PAMP-triggered immunity) (Nicaise et al., 2009). PTI aims to block further colonization by inducing changes in ion fluxes across plasma membranes, releasing apoplastic reactive oxygen species (ROS) and cytosolic calcium influx, activation of mitogen-activated protein kinases (MAPKs) followed by the production of phytohormones [e.g., ethylene and salicylic acids (SAs)], stomatal closure, callose deposition, cell wall reinforcement, accumulation of phytoalexins, and transcriptomic/metabolic reprogramming (Jones and Dangl, 2006; van Loon et al., 2006; Saijo et al., 2018; Wang et al., 2020). As an evolutionary result, pathogens adapted to suppress PTI by secreting effectors (virulence factors) into the apoplast (the host–pathogen interface) or within the cytoplasm, resulting in effector-triggered susceptibility. This leads to the second plant-defensive layer: Effector-triggered immunity (ETI). The host recognizes the effectors by nucleotide-binding leucine-rich repeat protein products encoded by resistance (*R*) genes (Jones and Dangl, 2006; Wang et al., 2020). ETI is a faster and amplified reboot of PTI, leading to disease resistance and hypersensitive reaction (HR) at the infection site. Pathogen–host co-evolution is a result of consecutive infection cycles that allowed pathogens and plants to diversify genes encoding for effectors and R proteins, respectively (Jones and Dangl, 2006). In addition to networks of inducible host defenses, plants also signal distant organs to become primed against further infection, known as systemic acquired resistance (SAR). The establishment of SAR involves systemic SA accumulation and the induction of pathogenesis-related (PR) proteins along with other defense genes [reviewed by Gao et al. (2015)].

Ink disease, or root rot, is caused by *Phytophthora cinnamomi* Rands, one of the most virulent and devastating plant pathogens worldwide. It infects more than 5,000 plant species and causes severe economic and ecological damage, making it one of the world’s top 10 most destructive oomycetes (Kamoun et al., 2015). It is a hemibiotrophic pathogen [reviewed by Hardham and Blackman (2018)] that begins the infection by the chemotactic attraction of the zoospores (asexual spores) to the roots of suitable hosts (Carlile, 1983). After penetrating the root, it rapidly reaches the vascular system, obstructing the xylem vessels and consequently impairing water and nutrient uptake (Oßwald et al., 2014).

The mechanisms of *Phytophthora* spp. infection have been mainly studied in the model species *P. infestans* and *P. sojae* (Jiang and Tyler, 2012) and represent a valuable source of data for *P. cinnamomi* research [reviewed by Hardham and Blackman (2018)]. The apoplast effectors secreted by *P. cinnamomi* can be cell wall–degrading enzymes (CWDEs), elicitins, toxins, and inhibitors of plant enzymes. Some cytoplasmic effectors have been identified as suppressants of host defense responses, such as callose deposition and HR cell death; however, most of their functions remain unknown. Some effectors are involved in disease establishment during zoospore adhesion and root penetration (e.g., CWDE). Others facilitate attack and colonization, guaranteeing *Phytophthora*’s access to nutrients for growth and reproduction [e.g., elicitins, transglutaminases, Crinkler (CRN), Nep1-like proteins (NLPs), RxLR proteins, and Avirulence (Avr) Nudix hydrolases; reviewed by Hardham and Blackman (2018)]. Some of these effectors and cell wall components (e.g., β-1,3- and β-1,6-glucans) are known as oomycete PAMPs; however, this knowledge is still limited (Raaymakers and Van Den Ackerveken, 2016). The dichotomy of PTI/ETI in *Phytophthora*–plant interactions is blurred, and recent studies indicate that these phases are more of a continuum and not distinct (Naveed et al., 2020).

*Phytophthora cinnamomi* has severely affected European chestnut (*Castanea sativa*) production and threatens its sustainability in forests. In Southwest Europe, control strategies have yet to be effective. So far, the best way to prevent further chestnut decline is mainly by planting first-generation Euro-Asian hybrids that inherited *P. cinnamomi* resistance from the co-evolved Asian host species, *Castanea mollissima* (Chinese chestnut) and *Castanea crenata* (Japanese chestnut) (Crandall et al., 1945; Serdar et al., 2019). This comes as a sustainable solution for production; however, it may impact the genetic diversity of European chestnuts in forests (Alcaide et al., 2022). Additional methods, such as biotechnological and/or phytopharmaceutical tools, should be developed to mitigate *P. cinnamomi*’s consequences and potentially reduce its prevalence. The key question remains unanswered: What causes some plant species to survive *P. cinnamomi* infection? During the last decade, significant advances were made in genomic research to understand plant–*P. cinnamomi* interactions by using approaches such as transcriptomics (Serrazina et al., 2015; Meyer et al., 2016; Reeksting et al., 2016; Baldé et al., 2017; Evangelisti et al., 2017; Islam et al., 2018; van den Berg et al., 2018b), genotyping by sequencing, association mapping, and identification of quantitative trait loci (QTL; Santos et al., 2015b, 2017b; Zhebentyayeva et al., 2019). Plant defense strategies against *P. cinnamomi* seem to vary in different hosts. For example, callose deposition is proposed as an important response for avocado (van den Berg et al., 2018a) and maize (Cahill and Weste, 1983), but it does not seem to play a significant role in chestnuts (Fernandes et al., 2021). However, chestnut species have similar mechanisms of defense against *P. cinnamomi* challenge. The European and Japanese chestnuts both induce genes related to the Jasmonic acid (JA) pathway, anti-fungal metabolite synthesis, anti-fungal enzymes, and cell wall strengthening (Serrazina et al., 2015). Also, association mapping studies of Euro-Japanese and American-Chinese *Castanea* hybrids inoculated with the pathogen reported two overlapping QTL regions, suggesting shared allelic variants (Santos et al., 2017b; Zhebentyayeva et al., 2019). The suggested difference between resistant and susceptible responses seems to be related to a high constitutive expression of putative resistance genes in the Japanese chestnut and a delay of pathogen-induced gene expression in the European chestnut (Santos et al., 2017a). The fast response by the resistant species favors the blockage of some infections and consequently reduces the area of infection (Fernandes et al., 2021). However, understanding the molecular architecture of plant–*P. cinnamomi* interactions remain unclear.

By comparing the root transcript profiles of resistant (*C. crenata*) and susceptible chestnut species (*C. sativa*) after infection with *P. cinnamomi*, this study aims to (i) identify pathogenicity-related genes expressed by *P. cinnamomi*, (ii) identify candidate genes linked with host resistance, and (iii) provide new insights into the molecular mechanisms involved in resistant and susceptible host defense responses. This study’s selected time points (2 h, 48 h, and 72 h after inoculation) represent specific stages of the pathogen’s infection and plant responses previously described by Fernandes et al. (2021). We used dual RNA-seq to reveal the complex interplay between *Castanea* spp. and *P. cinnamomi*, as it allows the temporal determination of responses and changes in both organisms’ cellular networks (Westermann et al., 2012). Also, the recent availability of the *P. cinnamomi* reference genome (Engelbrecht et al., 2021) has revealed effectors that may have a role in chestnut invasion and, consequently, facilitate the identification of corresponding putative *R* genes for future validation. We also took advantage of the availability of optimized *in vitro* propagation protocols (Fernandes et al., 2020) and produced biological replicates for each condition, which were individually sequenced. The results suggest new *Castanea* genes involved in pathogen perception and activation of immune responses and set forward *P. cinnamomi* genes that could have pathogenicity roles in the interaction with chestnuts.

## 2 Materials and methods

### 2.1 Plant material, pathogen inoculation, and sampling

Plants of *Castanea sativa* (CS12) and *Castanea crenata* (CC14) (genotypes from TRAGSA-SEPI, Maceda, Spain) were produced as described by Fernandes et al. (2020) and grown in a peat:perlite:vermiculite (1:1:1) mixture for 3 months. The roots were carefully washed with water before inoculation to remove the substrate. Roots were inoculated with *Phytophthora cinnamomi* mycelium (GenBank, accession number OL901253; isolate IMI 340340 from the University of Trás-os-Montes and Alto Douro). Pathogen growth conditions and inoculation methods followed the described by Fernandes et al. (2021). Briefly, *P. cinnamomi* cultures were grown in vegetable juice (V8^®^) agar [20% (v/v) with CaCO_3_ (3 g/L) and agar (6 g/L)] in darkness at 24°C for 5 days. Each plant was placed into a container with a paper base and a cotton ball moistened with sterile distilled water to avoid desiccation. Several pieces of actively growing mycelium were placed, covering the roots. Approximately 80% of the root system was inoculated as the roots next to the collar were forced upward, preventing the inoculation in that area.

Based on previous microscopic analysis (Fernandes et al., 2021), three time points were selected, corresponding to key stages of *P. cinnamomi* infection and plant responses. (i) 2 h after inoculation (hai): Pathogen’s penetration and establishment of biotrophic growth in susceptible and resistant species; beginning of defense responses in resistant species (callose around intracellular hyphae, accumulation of phenolic-like compounds in the cell walls and cytoplasmic contents, and hypersensitive response). (ii) 48 hai: Biotrophic pathogen growth in both species, with a delay detected in the progression of infection in the resistant species, associated with lower areas of hyphae colonization and higher development of the defense responses compared to the susceptible species (where occasionally callose around some intracellular hyphae was observed). (iii) 72 hai: Switch to necrotrophic phase and formation of survival structures in the susceptible species, in contrast with more restricted pathogen growth areas in the resistant species, associated with defense responses. Three plants were inoculated, and three were non-inoculated (control) per time point. In total, nine inoculated and nine non-inoculated plants for each chestnut species (36 plants total) were used. Plants were maintained at 24°C at a natural photoperiod.

### 2.2 Light microscopic observation of fresh tissues

Light microscopic observations were performed according to Fernandes et al. (2021) to guarantee that sequenced samples were infected and time points corresponded to the previously described stages of infection. Segments of approximately 2 mm to 5 mm in length were randomly collected from inoculated and non-inoculated root tissue at the referred time points. Samples were fixed in a 1:1:18 FAA solution—formaldehyde (37% to 38%)/glacial acetic acid/ethanol (70%)—and stored at 4°C until used. Root segments were rehydrated with distilled water for 15 min before sectioning (20-µm to 25-µm thick) in a freezing microtome (Leica CM1850). Longitudinal root sections were stained and mounted in cotton blue lactophenol (Silva et al., 2002). Observations were performed using a Leica DM-2500 light microscope equipped with a LEICA DFC340FX camera. Images were acquired with the software Leica Application Suite Version 4.12 (Leica Microsystems, Switzerland).

### 2.3 RNA extraction, quantification, and quality assessment

Total RNA was extracted individually for all 36 root samples as described by Le Provost et al. (2007). All samples were treated with the Turbo DNA-free™ Kit (Ambion, Life Technologies, Ltd.) according to the manufacturer’s instructions. Total RNA concentration and purity were verified with a NanoDrop spectrophotometer ND-2000C (Thermo Scientific) by measuring absorbance at 260/280 and 260/230. Concentration was verified with Qubit 2.0. The integrity was checked by electrophoresis on 1% agarose gel (1× TBE), and the absence of genomic DNA was confirmed by PCR, using primers for a known gene in both species [*Cast_Gnk2-like* gene; primers and PCR conditions are described by McGuigan et al. (2020)].

### 2.4 mRNA Library preparation and sequencing

RNA was sequenced by NOVOGENE Co. (Cambridge, UK). The NEBNext^®^ Ultra™ Directional RNA Library Prep Kit for Illumina^®^ was used to prepare 36 strand-specific cDNA libraries. mRNA was purified from total RNA using oligo(dT) magnetic beads, and rRNA was removed using the Ribo-Zero kit. First, the mRNA is fragmented randomly by adding fragmentation buffer, and, then, the cDNA is synthesized by using mRNA template and random hexamers, after which a custom second-strand synthesis buffer (Illumina), deoxynucleotide triphosphates (dNTPs), ribonuclease (RNase) H, and DNA polymerase I are added to initiate the second-strand synthesis. Second, after a series of terminal repair, A ligation, and sequencing adaptor ligation, the double-stranded cDNA library is completed through size selection and PCR enrichment. Quality control of libraries was performed. The concentration was checked on a Qubit 2.0 fluorometer (Life Technologies), the insert size was checked on an Agilent 2100, and the quantification was performed through qPCR. Stranded libraries were sequenced on Illumina NovaSeq 6000 platform following the manufacturer’s recommendations, and approximately 67 million paired-end strand-specific reads of 150 bp were produced per sample.

### 2.5 *Phytophthora cinnamomi* differential gene expression analysis

RNA-seq read quality before and after trimming was assessed using FastQC v0.11.9 (https://www.bioinformatics.babraham.ac.uk/projects/fastqc/), and results were aggregated with MultiQC v1.11 (Ewels et al., 2016). Low-quality reads were filtered, and adapters were removed using Trimmomatic v0.39 (Bolger et al., 2014). Reads with less than 50 bp were removed, and reads with an average Q score below 20 were trimmed. Trimmed reads were aligned to the *Phytophthora cinnamomi* reference genome (Engelbrecht et al., 2021) using STAR aligner v2.7.9a (Dobin et al., 2013). Unmapped reads were collected for *de novo* assembly transcriptomes of both *Castanea* species. Counts per gene were summarized using featureCounts in the SubReads package v2.0.2 (Liao et al., 2014). Lowly expressed genes were filtered, and the resulting dataset was balanced following the trimmed mean of M-value method (Robinson and Oshlack, 2010) implemented in edgeR v3.34.0 (Robinson et al., 2010). Depth and gene length were normalized transforming pair-reads to fragments per kb per million counts with rpkm function. After dispersion between samples was evaluated, pairwise comparisons were performed with the exactTest function. Obtained p-values were re-adjusted (by the Benjamini–Hochberg procedure), and the differently expressed genes (DEGs) were filtered out by false discovery rate (FDR) of ≤0.05 and log2 fold change of ≥1.0 or ≤–1.0.

### 2.6 *De novo* assembly of *Castanea sativa* and *Castanea crenata* transcriptomes

Due to the absence of a reference genome of *Castanea sativa*, *de novo* transcriptome assembly of both *Castanea* species was performed using Trinity v2.12.0 (Grabherr et al., 2011) with the unmapped reads obtained previously. The representative isoform was selected as the longest isoform using the “get_longest_isoform_seq_per_trinity_gene.pl” utility in Trinity. Contigs with less than 250 bp were excluded. Weakly expressed contigs were also removed on the basis of their expression values (Haas et al., 2013) using the Trinity script “aling_and_estimate_abundance.pl” to estimate transcripts per million (TPM) values using Salmon (Patro et al., 2017) and then using “filter_low_expr_transcripts.pl” Trinity utility to exclude lowly expressed contigs (–min_expr_any 0.5). The transcriptome assembly was annotated using dammit! pipeline v1.2 (Scott, 2016), which was based on Pfam-A, Rfam, OrthoDB, and *Castanea mollissima* protein (GCA_000763605.2) databases. Contigs were taxonomy annotated using MG-RAST v4.0.3 (Meyer et al., 2008) with default parameters, and contigs assigned with Streptophyta phylum were retained. Benchmarking Universal Single-Copy Orthologs (BUSCO) v5.2.2 (Manni et al., 2021) was used to evaluate the assembly completeness using the embryophyta_odb10 database. To assess the read content of the assembly, reads were mapped back to the transcriptome assembly using Bowtie2 v2.4.5 (Langmead et al., 2019). Assembly statistics were obtained with Quast v5.0.2 (Mikheenko et al., 2018). *Castanea* spp. differential gene expression analysis was performed as previously for *Phytophthora cinnamomi*. DEGs were blasted against NCBI non-redundant (nr) protein database with Blast2GO v6.0.3 (Gotz et al., 2008), using a cutoff e-value of 1E−5 and an HSP-hit coverage of 80%. Blast2GO was also used to assign Kyoto Encyclopedia of Genes and Genomes (KEGG) metabolic pathways (Kanehisa and Goto, 2000). Genes putatively related to disease resistance were annotated with Disease Resistance Analysis and Gene Orthology (DRAGO 3) tool based on the Plant Resistance Genes database (García et al., 2022). OrthoFinder v2.5.4 (Emms and Kelly, 2019) was used to predict orthologs within both *Castanea* species. Differential gene expression analysis was performed as in the previous section.

### 2.7 Functional enrichment analysis

The lists of DEGs from *Phytophthora cinnamomi* and *Castanea* species were analyzed with enricher function from clusterProfiler v4.2.2 package (Wu et al., 2021) to identify functional categories significantly enriched according to Gene Ontology (GO) terms identified using Pannzer2 (Törönen et al., 2018). For *Castanea* species assemblies, protein sequence was translated from the transcriptome assembly with TransDecoder v5.5.0 (https://github.com/TransDecoder/TransDecoder). Enricher function uses a hypergeometric test, and the obtained Gene Ratio is defined as the ratio of the number of DEGs and total gene number in specific GO terms. Significantly enriched categories were selected considering the adjusted p-value ≤ 0.05 (Benjamini–Hochberg correction for multiple testing).

## 3 Results

All plants were alive before the root samples were collected for RNA extractions and histopathology. Slight root browning and oxidation were generally observed, but there were no signs of necrotic lesions. The efficacy of inoculation was verified microscopically by the presence of hyphae in the root tissue and by observing the progression of the pathogen’s infection stages (**Supplementary Figure 1**).

### 3.1 *Phytophthora cinnamomi* cellular progression of infection

**Figure 1:**
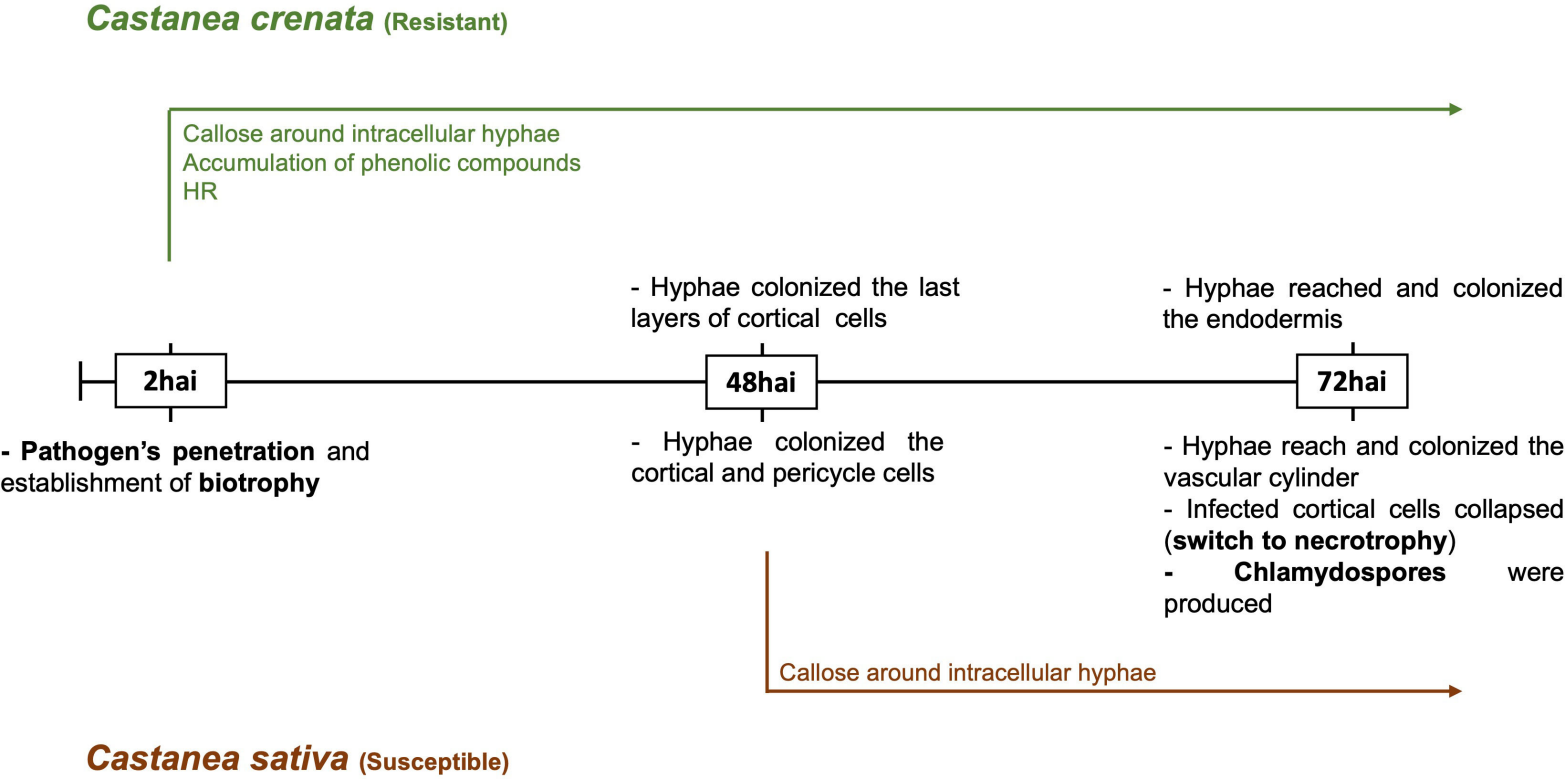
Time course of *Phytophthora cinnamomi* infection in roots of resistant and susceptible chestnut species. hai, hours after inoculation. The resistance was characterized by more restricted pathogen growth associated with the early activation of defense responses, such as callose around intracellular hyphae, accumulation of phenolic compounds in the cell walls and cytoplasmic contents, and the hypersensitive reaction (HR). In susceptible roots, callose deposition around some intracellular hyphae, observed from 48 hai, did not prevent the pathogen growth Fernandes et al. (2021).

The observed progress of infection followed the previously described by Fernandes et al. (2021). *Phytophthora cinnamomi* penetrated the root surface and continued to develop post-penetration in both chestnut species (**Supplementary Figure 1**). The switch to necrotrophy was observed in the susceptible *Castanea sativa* (**Supplementary Figure 2**). **Figure 1** describes the observed infection process at each time point in both species and the previously described early-induced host responses (Fernandes et al., 2021).

### 3.2 RNA sequencing and transcriptome assembly

Approximately 33 million read pairs were obtained per sample, and 14.7% and 22.55% were mapped to *Phytophthora cinnamomi*’s genome in the *Castanea crenata* and *Castanea sativa* inoculated samples, respectively (**Supplementary Table 1**). A few reads from the control samples were mapped to *P. cinnamomi* genome (0.001%; **Supplementary Table 1**), which were considered conserved eukaryotic gene sequences. After filtering low-expression transcripts, 19,981 genes were annotated in *P. cinnamomi* transcriptome (**Supplementary Table 2**). The number of *P. cinnamomi* transcripts expressed during each condition (chestnut species and time point) is provided in **Supplementary Table 2**.

*Castanea* spp. *de novo* transcriptome assembly produced 98,389 and 95,820 transcripts for *C. sativa* and *C. crenata*, respectively. For further analysis, 14,733 and 15,314 transcripts for *C. sativa* and *C. crenata*, respectively, were retained after being taxonomically annotated to the Streptophyta phylum (**Supplementary Table 3**).

### 3.3 *Phytophthora cinnamomi* differential expression analysis in *Castanea crenata* vs. *Castanea sativa* highlighted enriched functions related to pathogen attack

**Figure 2:**
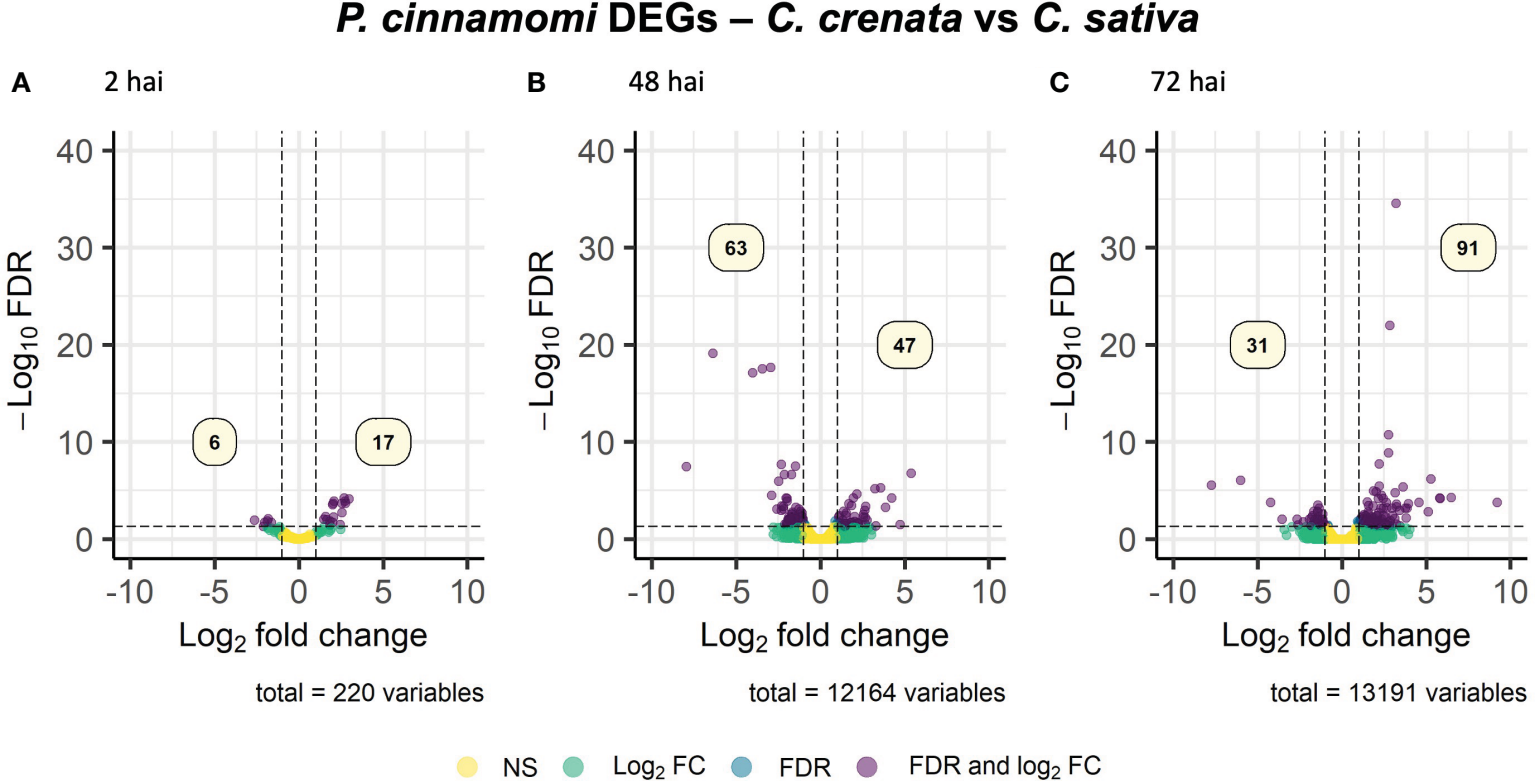
Volcano plots showing the dispersion of *Phytophthora cinnamomi* differentially expressed genes (DEGs) in *C. crenata* roots compared to *C. sativa* at 2 **(A)**, 48 **(B)**, and 72 **(C)** hours after inoculation (hai). DEGs were filtered out by false discovery rate (FDR) of ≤0.05 and log2 fold change (Log2 FC) of ≥1.0 or ≤–1.0. NS, not significant.

**Figure 3:**
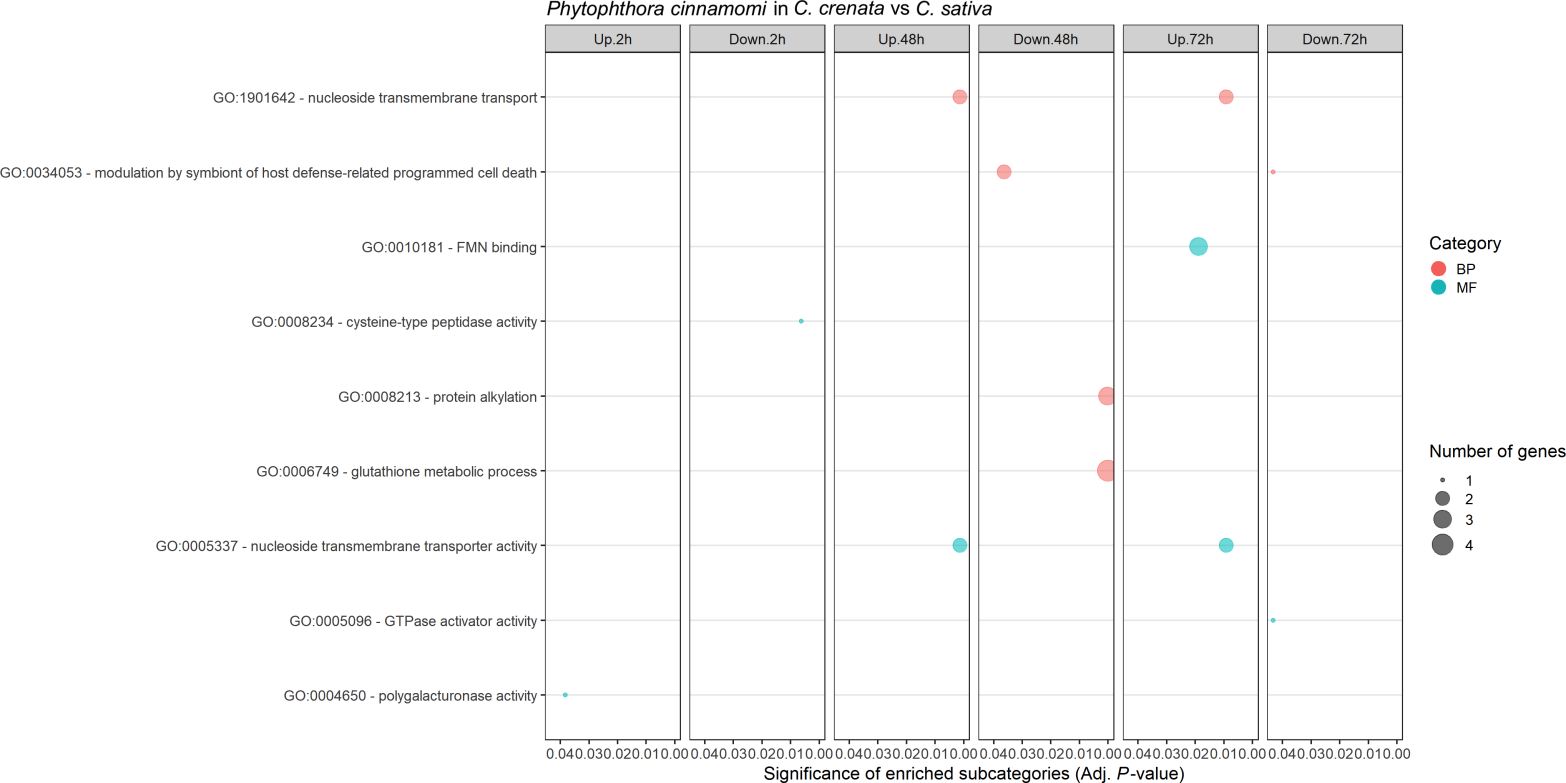
Gene Ontology (GO) enriched subcategories of *Phytophthora cinnamomi* differentially expressed genes in *Castanea crenata* roots compared with *Castanea sativa*, at 2 h, 48 h, and 72 h after inoculation. Analysis was performed using the hypergeometric statistical test, and significantly enriched categories were selected based on the adjusted P-value ≤ 0.05; BP, biological process, red circles; MF, molecular function, green circles. The complete dataset is provided in **Supplementary Table 5**.

*Phytophthora cinnamomi* transcript expression in *Castanea crenata* roots was compared to the expression in *Castanea sativa* roots at each time point after inoculation (**Supplementary Table 4**). The Multi-Dimensional Scaling (MDS) plots showed a good separation between samples at each time point (**Supplementary Figure 3**). In this analysis, upregulated genes correspond to *P. cinnamomi* genes that were more expressed in *C. crenata* roots than in *C. sativa*. Downregulated genes were genes less expressed in *C. crenata*, therefore more expressed in *C. sativa* roots. A total of 236 DEGs were identified, with the highest number of down- and upregulated genes occurring at 48 hai and 72 hai, respectively (**Figure 2**). No DEGs were common to all the time points (**Supplementary Figure 4**). Nine GO terms were enriched, most of them at 48 hai and 72 hai (**Figure 3**; **Supplementary Table 5**). Several GO terms related to pathogenicity, such as *cysteine-type peptidase activity* (2 hai) and *modulation by symbiont of host defense-related programmed cell death* (48 hai and 72 hai) were enriched in *C. sativa* samples (downregulated) and *polygalacturonase activity* (2 hai) in *C. crenata* (upregulated; **Figure 3**).

**Figure 4:**
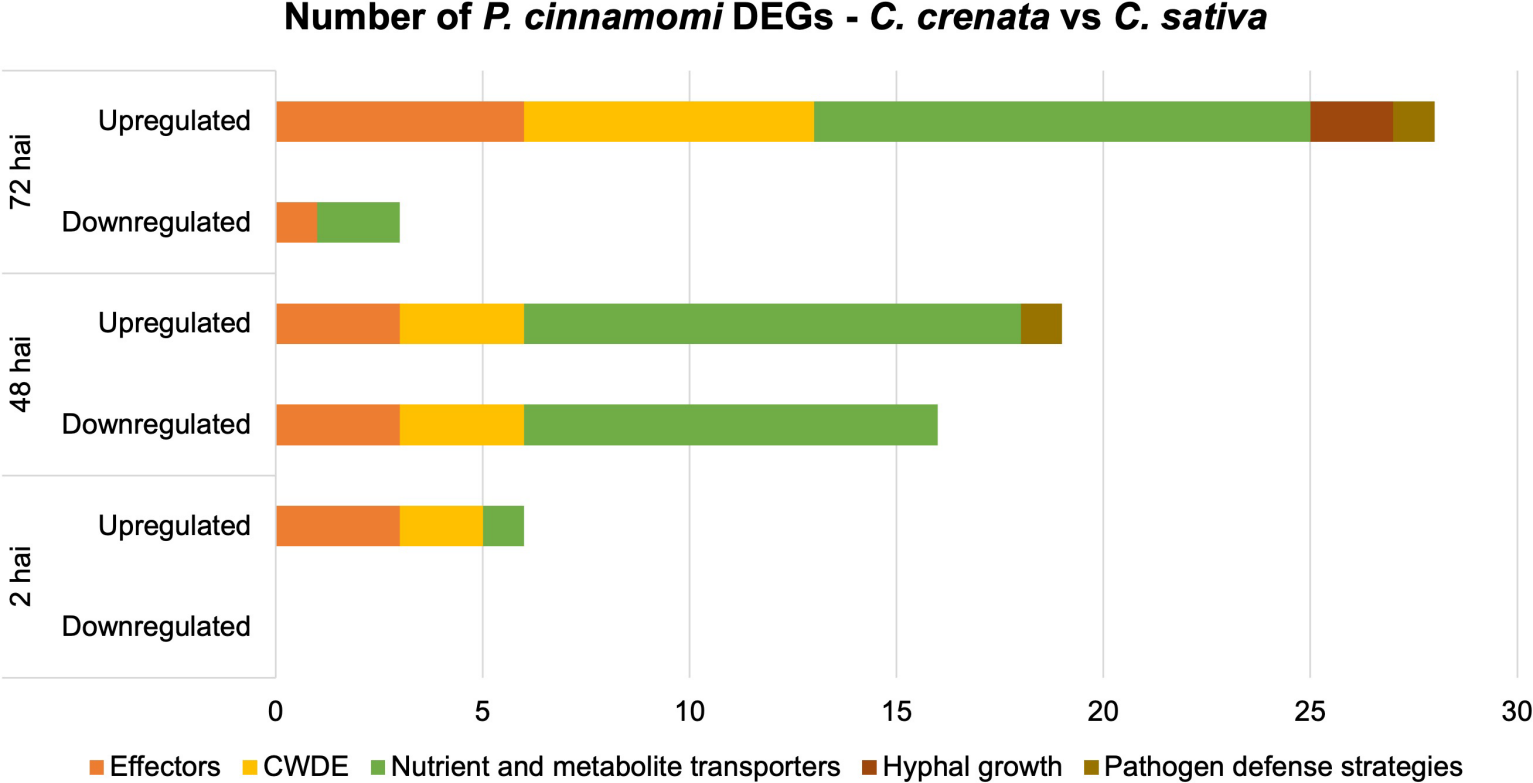
Number of differentially expressed genes (DEGs) by *Phytophthora cinnamomi* at 2 h, 48 h, and 72 h after inoculation (hai) in *Castanea crenata* roots compared to *Castanea sativa*. The selected DEGs represent several functional groups related to pathogen infection: effectors of pathogen attack, cell wall–degrading enzymes (CWDEs), nutrient and metabolite transporters, hyphal growth, and putative effectors of pathogen defense strategies. Upregulated: *P. cinnamomi* genes more expressed in *C. crenata* than in *C. sativa* roots. Downregulated: *P. cinnamomi* genes less expressed in *C. crenata* than in *C. sativa* roots and, therefore, more expressed in *C. sativa*.

Several transcripts encoding for *P. cinnamomi* effectors, such as proteins associated with pathogenicity, plant colonization, and transporters for organic compounds (e.g., sugars, amino acids, and nucleosides), ammonium, and phosphate were differentially expressed (DE) and selected for further analysis. Transporter selection was performed according to Abrahamian et al. (2016). A total of 15 effectors of pathogen attack strategies, 14 CWDE, 30 transporters, and two genes involved in hyphal growth were selected (**Figure 4**; **Supplementary Table 6**). Also included were two genes annotated for putative effectors of pathogen defense strategies, Kazal-like serine protease inhibitor and Catalase upregulated at 48 hai and 72 hai, respectively (**Supplementary Table 6**). Overall, there is an exponential increase throughout the time points of the number of *P. cinnamomi* genes more expressed in *C. crenata* tissues, unlike the downregulated genes (genes more expressed in *C. sativa* roots), which have a peek at 48 hai and a drastic decrease at 72 hai (**Figure 4**).

At 2 hai, genes encoding for three effectors of pathogen attack, two CWDEs, and one transporter were upregulated (**Figure 4**). The upregulated effectors were *Transglutaminase elicitor M81C*, *putative suppressor of necrosis 1*, and *Secretory protein OPEL* (**Supplementary Table 6**). At 48 hai, all the DE CWDEs were mainly from the hydrolase family, but the type of effectors of pathogen attack and transporters differed between up- and downregulated genes. At this time point, downregulated effectors were primarily elicitins, and upregulated were *Aldose 1-epimerase*, *Avr1b-1*, *RxLRs*, and *Crinkler (CRN)–like*. Ammonium and sugar transporters were found to be upregulated, and phosphate, sodium, and amino acid/auxin permeases *(AAAP)* were mostly downregulated (**Supplementary Table 6**). At 72 hai, two transcripts encoding for Mucin-like proteins were upregulated in *C. crenata* tissues by 4.5 and 9.2 fold change compared to those in *C. sativa*.

For further insight into the differences in *P. cinnamomi* gene expression in both chestnut species, we assessed the top 10 most up- and downregulated genes at each time point with known or putative functions in pathogenicity, growth, and sporulation (**Supplementary Table 7**). Twenty-five of the predicted proteins show homology to transcripts with known functions in virulence and pathogenicity based on functional genetic assays performed with other pathogens. Twelve of these were not included in the assessment represented in **Figure 4**. Additionally, two downregulated transcripts related to *Phytophthora* zoospore development were identified: *DEAD/DEAH box RNA helicase* and *Zinc finger*, *C2H2* (**Supplementary Table 7**).

### 3.4 *Phytophthora cinnamomi* differential gene expression at 72 h vs. 48 h after inoculation reveals different enriched functions in *Castanea sativa* and *Castanea crenata*

**Figure 5:**
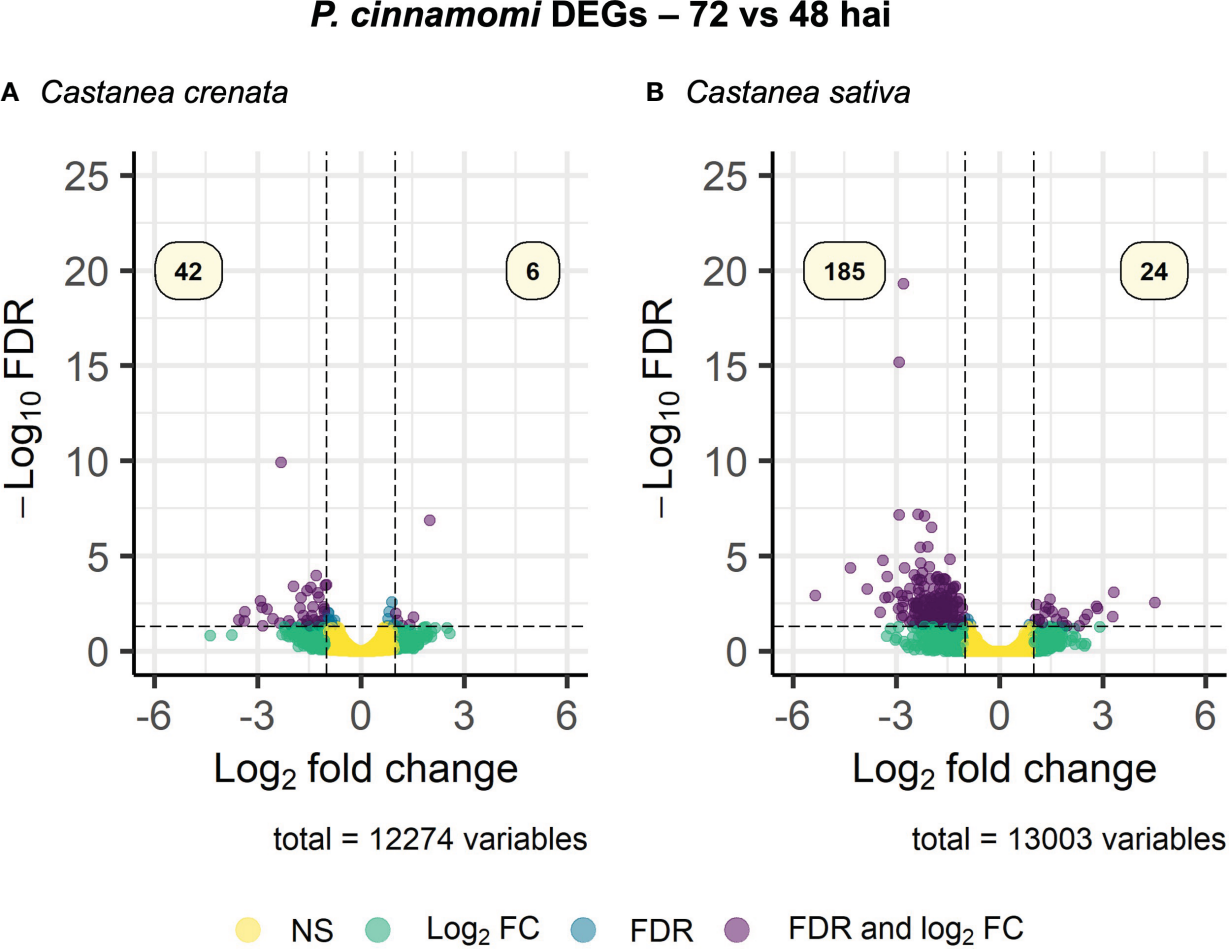
Volcano plot showing the dispersion of *Phytophthora cinnamomi* differentially expressed genes (DEGs) in *Castanea crenata* **(A)** and *Castanea sativa* **(B)** roots at 72 h after inoculation (hai) compared with 48 hai. DEGs were filtered out by false discovery rate (FDR) of ≤0.05 and log2 fold change (Log2 FC) of ≥1.0 or ≤–1.0. NS, not significant.

**Figure 6:**
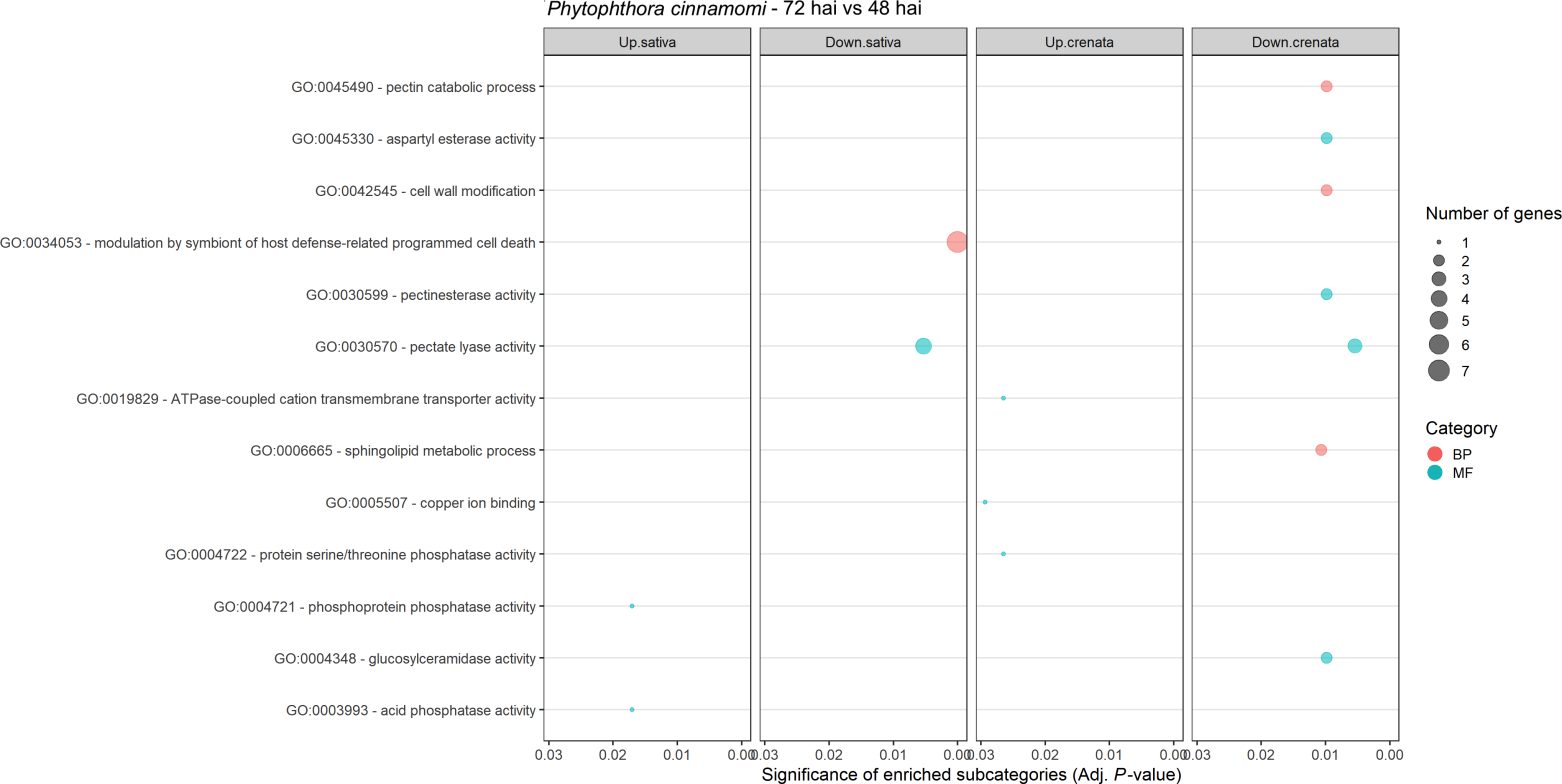
Gene Ontology (GO)–enriched terms of *Phytophthora cinnamomi* differentially expressed genes at 72 h after inoculation (hai) when compared to 48 hai in *Castanea crenata* and *Castanea sativa* roots. Analysis was performed using the hypergeometric statistical test, and significantly enriched categories were selected based on the adjusted p-value ≤ 0.05; BP, biological process, red circles; MF, molecular function, green circles. The complete dataset is provided in **Supplementary Table 9**.

Expression of *Phytophthora cinnamomi* transcripts infecting *Castanea crenata* and *Castanea sativa* at 72 hai was compared to that at 48 hai. The MDS plots showed a good separation between the samples of the different time points for each species (**Supplementary Figure 5**). A total of 237 DEGs were identified, 209 in *C. sativa*, and 48 in *C. crenata* (**Supplementary Table 8**). Most of the DEGs were found to be downregulated at 72 h vs. 48 h in *C. sativa* roots (**Figure 5**). Nineteen downregulated genes were common to both species (**Supplementary Figure 6**). Four GO terms were enriched in *C. sativa* and 10 in *C. crenata* (**Figure 6**; **Supplementary Table 9**). GO terms related to pathogen attack were mainly identified in downregulated genes (genes more expressed at 48 hai) in both species. In *C. sativa*, the enriched terms were *modulation by symbiont of host defense-related programmed cell death* and, *pectate lyase activity.* In *C. crenata*, GO terms were mostly related to host cell wall degradation (*pectin catabolic process*, *aspartyl esterase activity*, and *pectinesterase activity*) and pathogen cell wall alteration (*cell wall modification*).

**Figure 7:**
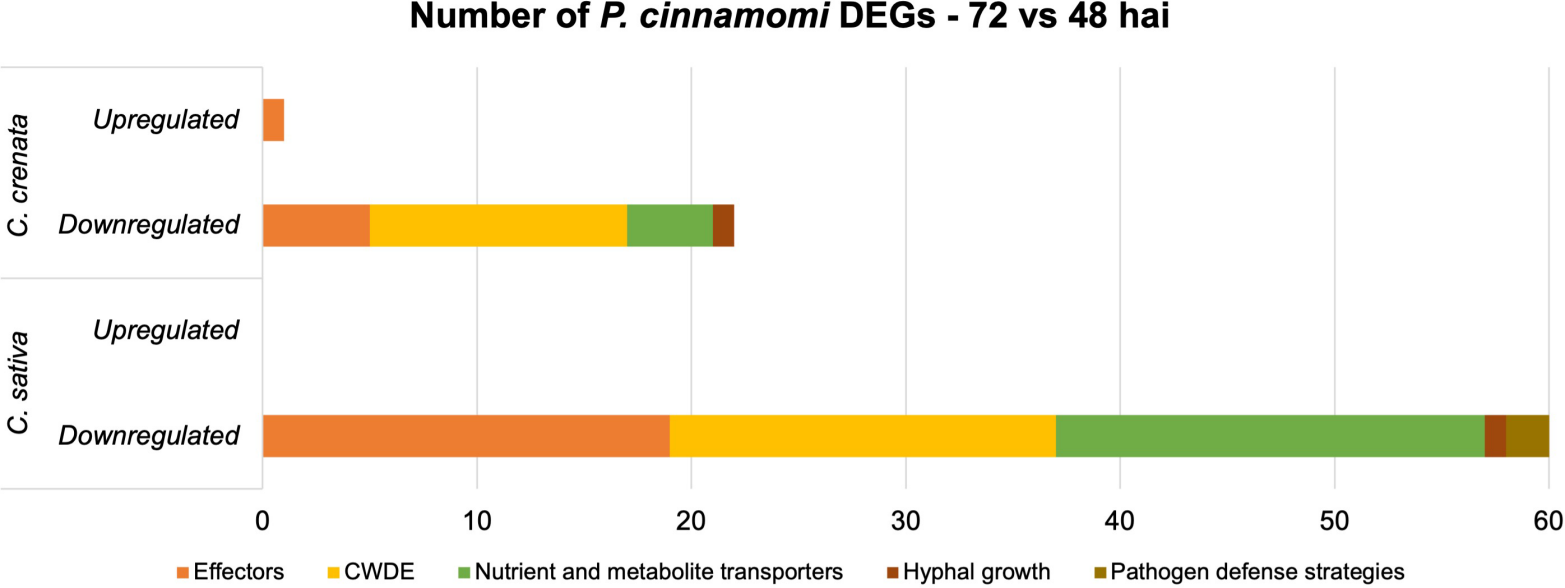
Number of differentially expressed genes (DEGs) expressed by *Phytophthora cinnamomi* 72 h after inoculation (hai) when compared to 48 hai in *Castanea crenata* and *Castanea sativa* roots. The genes represented in this graph are related to pathogen development and infection: effectors of pathogen attack, cell wall–degrading enzymes (CWDEs), nutrient and metabolite transporters, hyphal growth, and putative effectors of pathogen defense strategies.

Genes for effectors of pathogen attack, CWDE, nutrient and metabolite transporters, hyphal growth, and putative effectors of pathogen defense strategies were selected for further analysis, as described in the previous section (**Supplementary Table 10**; **Figure 7**). Only one gene was upregulated, corresponding to an effector of pathogen attack in *C. crenata* (**Supplementary Table 10**). Overall, the pathogen downregulated more genes at 72 hai in *C. sativa* compared to *C. crenata*, most being effectors and nutrient and metabolite transporters (**Figure 7**). This includes several *Necrosis-inducing like proteins*, *Elicitins*, *RxLRs*, *Transglutaminase elicitor M81C*, and *putative suppressor of necrosis 1*. The higher fold-change DEGs were *Aldose 1-epimerase* and *Avr4-associated TDF-like protein* with −3.84 and −3.21, respectively (**Supplementary Table 10**). From the 20 DE transporters identified in *C. sativa*, the most common were *Amino Acid/Auxin Permease* (*AAAP*) *Family* and *Major Facilitator Superfamily* (*MFS*) (**Supplementary Table 10**). Also downregulated in *C. sativa* roots were *Catalase/peroxidase HPI* and *protease inhibitor Epi11*, related to pathogen defense strategies (**Supplementary Table 10**).

We also looked into the top 10 most up- and downregulated in each species with known functions and/or with hits (coverage > 80%; *E-*value < 1E−3) in the PHI-BASE with the same selection criteria described in the previous section. However, all genes that fitted the criteria were already included in **Supplementary Table 10**, except for Transcript IUM83_01168, downregulated in *C. crenata*. This encodes for a pleiotropic drug resistance protein ABC superfamily (KAG6618853.1), which had a hit in the PHI-BASE with *GPABC1* (Entry: 258; E-value: 1.37E−158), also an ABC transporter, that, when knocked down, reduces pathogen virulence.

### 3.5 Differentially expressed genes highlighted specific enriched functions in resistant and susceptible chestnuts after pathogen attack

Differential expression analysis in chestnut transcriptomes was performed by comparing inoculated with non-inoculated plants at three time points after inoculation. MDS plots showed separation between control and inoculated samples at all time points. This suggests that a *Phytophthora cinnamomi* infection influences *Castanea sativa* and *Castanea crenata* transcriptomic profiles, enabling discrimination between the control and inoculated samples (**Supplementary Figure 7**).

**Figure 8:**
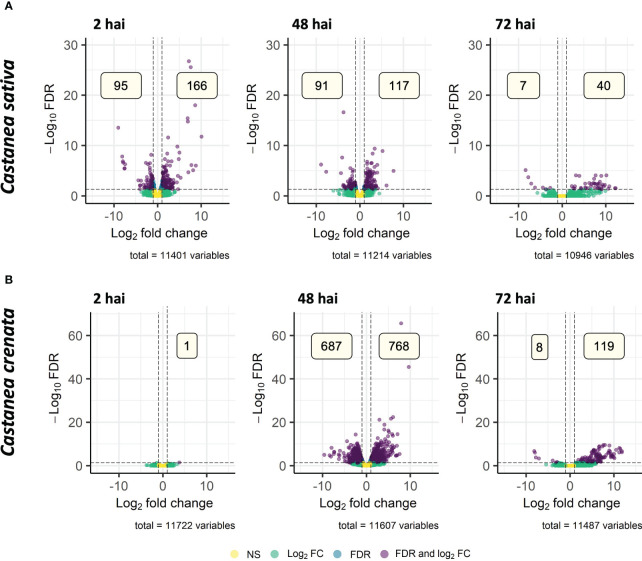
Volcano plots showing the dispersion of differentially expressed genes (DEGs) of *Castanea sativa* **(A)** and *Castanea crenata* **(B)** inoculated with *Phytophthora cinnamomi* when compared to non-inoculated controls at 2 h, 48 h, and 72 h after inoculation (hai). DEGs were filtered out by false discovery rate (FDR) of ≤0.05 and log2 fold change (Log2 FC) of ≥1.0 or ≤–1.0. NS, not significant.

From 14,733 (*C. sativa*) and 15,314 (*C. crenata*) transcripts mapped for Streptophyta phylum, 488 (*C. sativa*) and 1,556 (*C. crenata*) were DE after *P. cinnamomi* inoculation, from which 406 and 1,293 had homology to *Castanea mollissima* protein database (**Supplementary Tables 11**, **12**). From all the DEGs, 188 and 667 of *C. sativa* and *C. crenata*, respectively, were annotated with Blastx. The remaining genes did not show a minimum of 80% similarity to any reported proteins used by this algorithm (**Supplementary Tables 11**, **12**); for that reason, the best dammit homology (lowest e-value < 1E−05) was considered. *Castanea sativa* upregulates the highest number of genes at 2 hai, and this number decreases in the subsequent time points (**Figure 8A**), whereas *C. crenata* with only one DE gene at 2 hai upregulates the highest number of genes at 48 hai, followed by a decrease at 72 hai (**Figure 8B**). Few DEG overlapped time points in both species (**Supplementary Figure 8**), and only Transcript_48468 was upregulated at all time points in *C. sativa* (**Supplementary Figure 8A**), which putatively encodes for a (+)-neomenthol dehydrogenase (**Supplementary Table 11**; dammit best homology).

**Figure 9:**
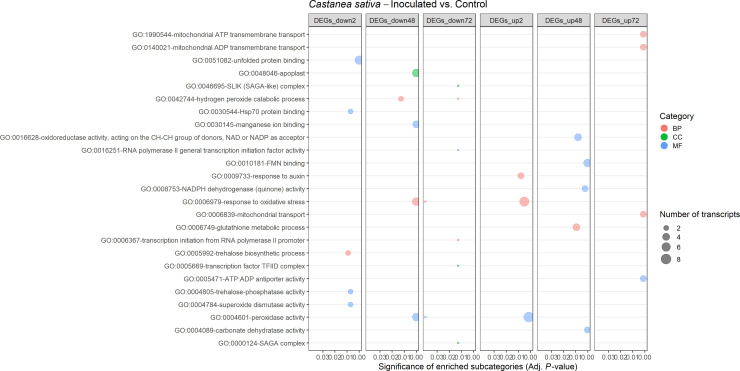
Gene Ontology (GO)–enriched terms in the differentially expressed genes of *Castanea sativa* at 2 h, 48 h, and 72 h after inoculation with *Phytophthora cinnamomi*. Analysis was performed using the hypergeometric statistical test, and significantly enriched categories were selected based on the adjusted p-value ≤ 0.05. Different categories are represented with different colors: red, biological process (BP); green, cellular component (CC); blue, molecular function (MF). The complete dataset is provided in **Supplementary Table 13**.

**Figure 10:**
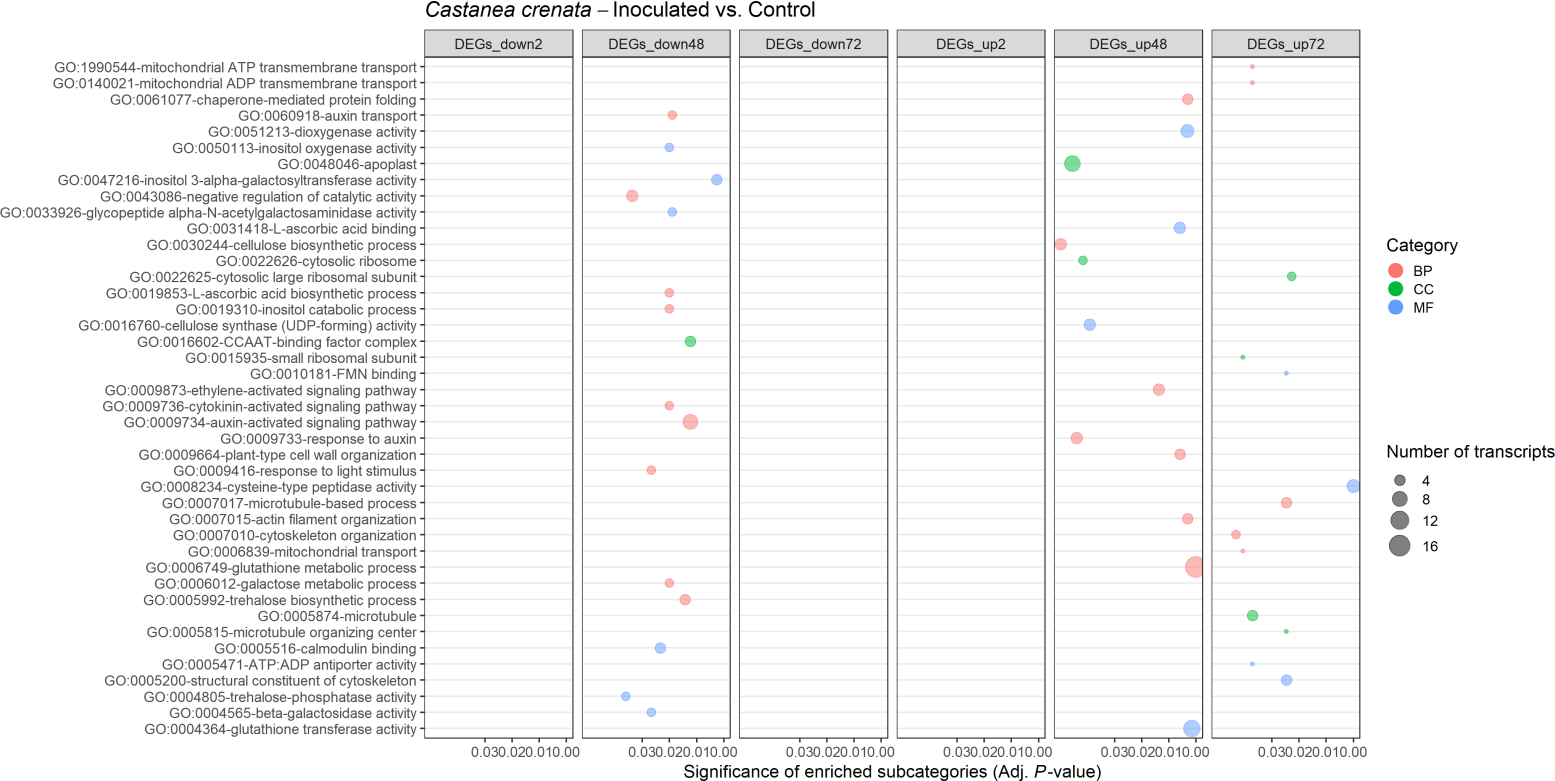
Gene Ontology (GO)–enriched terms in the differentially expressed genes of *Castanea crenata* at 2 h, 48 h, and 72 h after inoculation with *Phytophthora cinnamomi*. Analysis was performed by using the hypergeometric statistical test and significantly enriched categories were selected based on the adjusted p-value ≤ 0.05. Different categories are represented with different colors: red, biological process (BP); green, cellular component (CC); blue, molecular function (MF). The complete dataset is provided in **Supplementary Table 14**.

A total of 25 and 42 GO terms were enriched in DEGs of susceptible (**Figure 9**) and resistant (**Figure 10**) chestnuts, respectively. Enriched GO terms related to biotic stress response, such as *apoplast*, *plant-type cell wall organization*, and *ethylene-activated signaling pathway*, were only enriched for *C. crenata* upregulated genes. Also, six GO terms related to cytoskeleton and microtubule organization were enriched in upregulated genes at 48 (*actin filament organization*) and 72 hai (*structural constituent of cytoskeleton*, *microtubule organizing center*, *microtubule*, *cytoskeleton organization*, and *microtubule-based process*) (**Figure 10**). *Response to auxin* was common to both species but at different time points (**Figures 9**, **10**), and several GO terms enriched in *C. sativa* were related to the balance of the redox homeostasis, such as *response to oxidative stress*, *peroxidase activity*, *superoxide dismutase activity*, and *hydrogen peroxide catabolic process*. However, most of these terms are associated with downregulated genes, and the first two mentioned GO terms are enriched in upregulated genes at 2 hai and then in downregulated genes at 48 hai and 72 hai (**Figure 9**).

**Figure 11:**
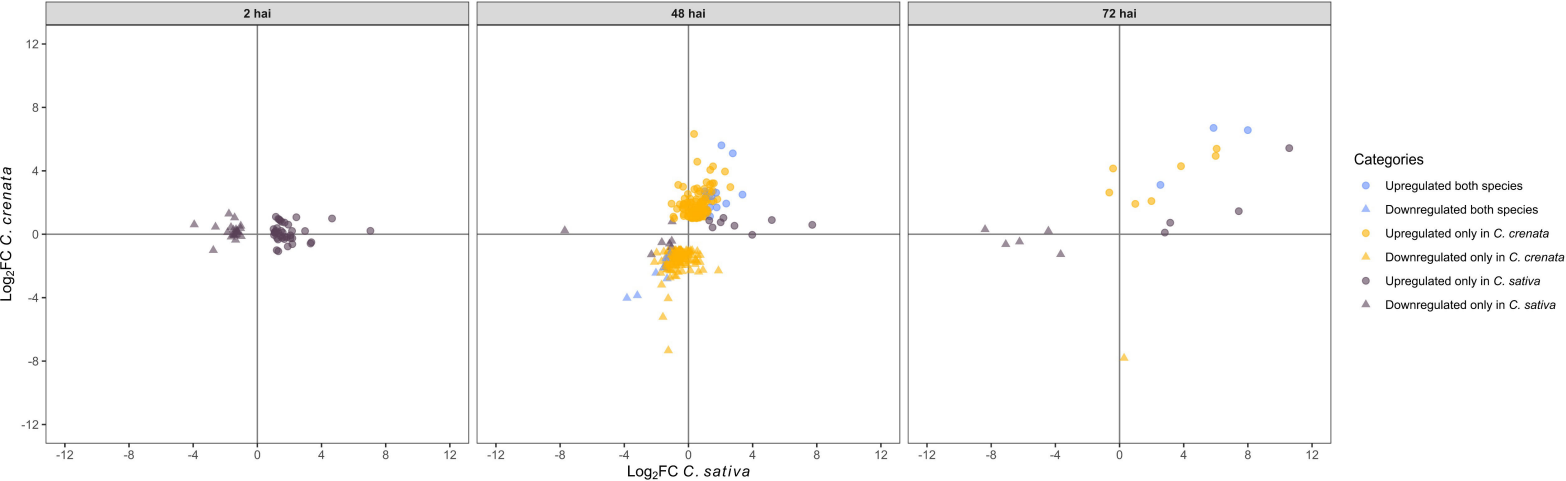
Scatterplot showing single-copy orthologs upregulated and downregulated in both species, only in *Castanea crenata* and only in *Castanea sativa* after inoculation with *Phytophthora cinnamomi*. DEGs were filtered out by false discovery rate (FDR) of ≤0.05 and log2 fold change (Log2 FC) of ≥1.0 or ≤–1.0.

OrthoFinder was used to identify orthologs within *Castanea* species (**Supplementary Tables 15**, **16**) and 515 DE single-copy orthologs (SCOs) were filtered and classified according to their expression at each time point (upregulated and downregulated in both species, only in *C. crenata*, and only in *C. sativa*; **Figure 11**; **Supplementary Table 17**). Two-hundred and fourteen SCOs were upregulated only in *C. crenata* (**Supplementary Table 18**).

### 3.6 *Castanea crenata* differentially expresses more putative resistance genes after inoculation compared to *Castanea sativa*

The analysis with Drago3 identified 35 and 7 DEGs from *Castanea crenata* and *Castanea sativa*, respectively, putatively encoding for resistance proteins with domains such as LRRs or kinase (Santana Silva and Micheli, 2020; Calle García et al., 2022; **Supplementary Tables 19**, **20**). Several ectodomains have been described as ligand-binding, such as LRR, LysMs, lectin-like motifs, and epidermal growth factor (EGF)–like domains (Saijo et al., 2018). An additional 43 and 3 DEGs for *C. crenata* and *C. sativa*, respectively, encoding for LRR, Kinase, LysM, and PAN-like domains were identified with the dammit algorithm. Of all the putative resistance genes identified for both species, several had corresponding orthologs, but only one was DE after inoculation (**Supplementary Tables 19**, **20**). Upregulated genes were considered candidate resistance genes. *Castanea crenata* upregulated 49 transcripts, among which 12 were SCOs exclusively upregulated in the resistant chestnut (highlighted in **Supplementary Table 19**); several putatively encode for PRRs such as G-type lectin S-receptor–like serine/threonine-protein kinase RLK1 (chitin receptor; Brotman et al., 2012), cysteine-rich RLP kinases, and leucine-rich repeat RLP kinase. Other genes encoding proteins involved in phosphorylation (serine/threonine-protein kinase STY13), PRR signal transmission (calcium-dependent protein kinase 10; Saijo et al., 2018), and protein degradation (26S proteasome regulatory subunit 4 homolog A; Adams and Spoel, 2018) were also exclusively upregulated by *C. crenata.* Multiple genes that did not have an ortholog in the susceptible species were also upregulated. Some examples putatively encode for the PRR chitin receptor Protein LYK5 (Cao et al., 2014), LRR receptor kinase BAK1, and the PRR signal transmission MAPK kinase kinase 17 (Saijo et al., 2018) (**Supplementary Table 19**). *Castanea sativa* upregulated only seven protein-coding genes, which also included PRRs (e.g., *Cysteine-rich RLP kinase 10*), MAPKs (*Mitogen-activated protein kinase 9*), and transporters (e.g., *Pleiotropic drug resistance protein 1*) (**Supplementary Table 20**). In both species, most genes are upregulated at 48 hai with the exception of *Cysteine-rich receptor-like protein kinase 10* at 2 hai in *C. sativa*, and *Rust resistance kinase Lr10*, *26S proteasome regulatory subunit 4 homolog A*, and a non-annotated gene in *C. crenata* at 72 hai (**Supplementary Tables 19**, **20**).

### 3.7 The induction of genes involved in the plant defense network was higher in *Castanea crenata*

Upregulated genes from both species with GO terms related to innate immune response, SAR, defense response, transcription factors, different phytohormone response and signaling pathways, cell wall, callose, calcium-binding, antifungal, chitin, and proteolysis were selected for analysis. Upregulated genes previously proposed as putatively involved in European and Japanese chestnuts’ susceptibility/resistance to *Phytophthora cinnamomi* [e.g., Santos et al. (2017a, 2017b), Serrazina et al. (2021), and Pavese et al. (2021)] that did not show in the previous selection were hand-searched. Selected genes were organized into categories related to their putative function suggested by the attributed GO terms and published literature and were further analyzed. A total of 41 and 90 genes were selected for *Castanea sativa* and *Castanea crenata*, respectively (**Tables 1**, **2**). Both species upregulated genes related to the induction of similar defense responses and signaling pathways such as elicitors of immune response (e.g., PAMP-induced secreted peptides), genes involved in recognition of Ca^2+^ signals stimulated by biotic stress and translation into cellular responses (calcium-binding proteins; Cheval et al., 2013), involved in SA, JA, and ET signaling regulation (e.g., ethylene-responsive transcription factors and SA-binding proteins), anti-fungal activity (e.g., thaumatin-like proteins), cell wall reinforcement (e.g., pectinesterases), lignin biosynthesis (e.g., dirigent protein-like), proteolysis (e.g., Cysteine proteinases), and oxidative stress response (e.g., peroxidases). However, *C. crenata* presents higher fold changes and upregulates more genes from all categories. Indeed, 25 selected genes were SCOs only upregulated in *C. crenata* such as *VQ motif-containing protein 1* and transcription factors (e.g., *bHLH162-like* and *NAC domain-containing protein*) (**Table 2**). Examples of genes induced by *C. crenata* that did not have orthologs in *C. sativa* include the PR proteins from families 2 (e.g., *glucan endo-1*,*3-beta-D-glucosidase-like*) and 4 (*pathogenesis-related protein PR-4-like*), the auxin transporter *auxin efflux carrier component 5* (*PIN5*) (Adamowski and Friml, 2015), and the HR promoter *Alpha-dioxygenase 1* (De León et al., 2002; **Table 2**). Some genes possibly related to susceptibility were identified in both species. In *C. sativa*, two expansins, which loosen the plant cell wall (Bellincampi et al., 2014) and the *auxin efflux carrier component 4* (*PIN4*) responsible for auxin concentration at the root tip (Adamowski and Friml, 2015), were upregulated at 2 hai; and *BON1-associated protein 2-like* (*BAP2-like*), an inhibitor of programmed cell death (Yang et al., 2007), was upregulated at 48 hai (**Table 1**). *Castanea crenata* also upregulated two expansins at 48 hai and 72 hai and *Downy mildew resistance 6* (*DMR6*) at 48 hai, a negative regulator of SA pathway (Pavese et al., 2021). However, *C. crenata* upregulated 11 chestnut genes currently proposed as candidates for *P. cinnamomi* resistance, including four SCOs uniquely upregulated in this species (**Table 2**). These genes putatively regulate defense responses (*transcription factor MYB4-like*; Santos et al., 2017a), induce pathogen osmotic rupture (*thaumatin-like protein 1*; Corredoira et al., 2012), secrete anti-fungal compounds (*Cysteine-rich repeat secretory protein 38*; Santos et al., 2017a), deposit pectates in the cell wall (*Pectinesterase 2*; Santos et al., 2017a), and synthesize cellulose in the cell wall (*Cellulose synthase-like proteins*; Santos et al., 2017b). Only one candidate resistance gene for *P. cinnamomi* resistance was identified in *C. sativa* (*thaumatin-like protein 1*; Corredoira et al., 2012; **Table 1**).

**Table 1:** *Castanea sativa* genes related to pathogen defense overexpressed at 2 h, 48 h, and 72 h after inoculation with *Phytophthora cinnamomi*.

Transcript CS	Fold change			Best homology	CC orthologs	Reference	Notes
	2 h	48 h	72 h				
Innate Immune Response							
Transcript_28492	1.8	3.38	NA	PAMP-induced secreted peptide 2	NA	Hou et al. (2014)	Immune response elicitor
Calcium-binding proteins							
Transcript_31903	NA	1.14	NA	Calcium-binding protein PBP1-like	NA	Zhang et al. (2014)	
Transcript_31927	NA	1.76	NA	Calcium-binding protein CP1	**Transcript_38648 (48 h)**		
Transcript_5726	1.97	NA	NA	Respiratory burst oxidase homolog protein B	NA	Kobayashi et al. (2007)	Pathogen-induced oxidative burst
Transcript_76190	NA	1.59	NA	CSC1-like protein ERD4	**Transcript_30714 (48 h)**	Rai et al. (2012)	Osmosensitive calcium-permeable cation channel
Defense response–related transcription factors							
Transcript_28684	1.17	NA	NA	Ethylene-response factor C3-like	NA	Lorenzo et al. (2003) Müller and Munné-Bosch (2015)	Possibly activated by ET/JA
Transcript_29638	1.65	NA	NA	Ethylene-responsive transcription factor 1	NA		
Transcript_64501	1.97	3.32	NA	Auxin-responsive protein IAA1	NA	Liscum and Reed (2002)	Repressors of early auxin response genes
Transcript_31337	1.85	NA	NA	Probable WRKY transcription factor 43	Transcript_37230	Eulgem et al. (2000)	
Transcript_75482	2.99	NA	NA	Probable WRKY transcription factor 74	Transcript_53497		
Transcript_39413	2.16	NA	NA	Protein RADIALIS-like 3	Transcript_31057	Yanhui et al. (2006)	MYB-related
Other defense response–related							
Transcript_60117	1.81	NA	NA	VQ motif-containing protein 1-like	NA	Yuan et al. (2021)	Interacts with WRKY and MAPKs
Transcript_41892	NA	2.41	NA	BON1-associated protein 2-like	NA	Yang et al. (2007)	Programmed cell death inhibitor
Transcript_9042	2.06	NA	NA	MLO-like protein 3	**Transcript_38690 (48 h)**	Kusch et al. (2019)	Possibly related to leaf senescence and pathogen defense (SAR)
Transcript_38325	NA	1.46	NA	Pathogenesis-related protein 1-like	**Transcript_43245 (48 h)** Transcript_33563	Ali et al. (2018)	Anti-fungal
Anti-fungal							
Transcript_35665	NA	2.32	NA	Thaumatin-like protein	**Transcript_29889 (48 h) Transcript_28076 (48 h)**	Ali et al. (2018) Corredoira et al. (2012)	Promotes osmotic rupture in pathogen
							Candidate resistance gene to *P. cinnamomi*
Transcript_30786	NA	2.62	NA	Acidic endochitinase Q	NA	Payne et al. (1990)	Chitinase activity
Cell wall rehinforcement/reorganization							
Transcript_33472	2.21	NA	NA	Expansin-a4	NA	Bellincampi et al. (2014)	Plant cell wall loosening/extension
Transcript_67012	2	NA	NA	Expansin-A3-like, partial	**Transcript_32643 (48 h)** **Transcript_32249 (48 h)** Transcript_42391		
Transcript_37840	1.4	NA	NA	Pectinesterase 3	NA	Kieffer et al. (2000)	Involved in *Phytophthora* spp. response
Apoplast							
Transcript_9167	NA	3.21	NA	Putative laccase-9	NA	Turlapati et al. (2011)	Probably involved in lignin polymerization
Transcript_29539	NA	1.75	NA	Dirigent protein 22-like	Transcript_55112 Transcript_27706 **Transcript_27146 (48 h)**	Paniagua et al. (2017)	Involved in lignin biosynthesis
Transcript_31686	1.3	NA	NA	Germin-like protein subfamily 2 member 4	Transcript_29603	Dunwell et al. (2008)	May play a role in pathogen defense
Proteolysis							
Transcript_30127	NA	2.77	NA	Metalloendoproteinase 2-mmp	Transcript_39339	Liu et al. (2001)	Up regulated after *Phytophthora* infection
Transcript_31868	1.95	NA	NA	Aspartic proteinase Asp1-like	NA	Balakireva and Zamyatnin (2018)	Related to biotic stress response May be involved in different plant immunity pathways (PTI, ETI, SAR)
Transcript_41998	1.19	NA	NA	Basic secretory protease	Transcript_92289 Transcript_83965		
Transcript_9290	NA	1.51	NA	Aspartyl protease family protein 2	NA		
Transcript_35737	NA	NA	8.37	Cysteine proteinase 3	Transcript_79258 **Transcript_50560 (72 h)**		
Phytohormone response and signaling pathways							
Transcript_34810	NA	3.98	NA	Late embryogenesis abundant protein B19.3	Transcript_36921	Hollung et al. (1994)	Osmoprotective protein
Transcript_9109	1.15	NA	NA	Salicylic acid-binding protein 2-like	**Transcript_38601 (48 h)**	Tripathi et al. (2010)	SAR. Converts MeSA to SA
Transcript_31113	1.91	NA	NA	Auxin-responsive protein SAUR78-like	Transcript_19889 **Transcript_43139 (48 h)**	Kant et al. (2009)	Negative regulation of auxin synthesis and transport
Transcript_32526	2.03	NA	NA	Auxin-responsive protein SAUR78-like			
Transcript_39048	2.01	2.43	NA	Auxin-responsive protein SAUR71-like	Transcript_38635 **Transcript_14360 (48 h)**		
Transcript_35330	1.84	NA	NA	Auxin efflux carrier component 4 PIN4	Transcript_93715	Adamowski and Friml (2015)	Role in creating a sink for auxin into columella cells
Oxidative stress response							
Transcript_44570	2.32	NA	NA	Hypothetical protein I3842_10G110600	NA		
Transcript_36951	1.12	NA	NA	Peroxidase 11	NA	Ali et al. (2018) Welinder et al. (2002) Alcázar et al. (1995) Bhai et al. (2021)	PR-9 Removal of H_2_O_2_ Involved in biosynthesis of lignin Related to *Phytophthora* spp. defense
Transcript_48474	1.12	NA	NA	Cationic peroxidase 1	NA		
Transcript_50879	2.36	2.79	NA	peroxidase 5	**Transcript_39196 Transcript_65197 (48 h)**		
Transcript_5676	1.21	NA	NA	Peroxidase 11	NA		
Transcript_66354	2.41	NA	NA	L-ascorbate peroxidase 3	NA		
Transcript_73393	1.64	NA	NA	Peroxidase N1	Transcript_53619		

Genes are divided into categories related to their Gene Ontology terms and putative functions. Best protein homology description (from blastx: cutoff e-value = 1E−5, HSP-hit coverage = 80%; or dammit: lowest e-value < 1E−05), and *Castanea crenata* (CC) ortholog transcripts are presented. Differentially expressed CC orthologs are in bold (upregulated) or bold and red (downregulated). Chestnut candidate resistance genes to *P. cinnamomi* are highlighted in blue. Genes putatively related to susceptibility are highlighted in orange. Darker shades of green represent higher values of positive log2FC (fold change). NA, not applicable; PTI, pathogen-associated molecular pattern (PAMP) triggered immunity; ETI, effector-triggered immunity; ET, ethylene; SA, salicylic acid; JA, jasmonic acid; MeJA, methyl jasmonate; ABA, abscisic acid; ROS, reactive oxygen species; MAPKs, mitogen-activated protein kinases; SAR, systemic acquired resistance.

**Table 2:** *Castanea crenata* (CC) genes related to pathogen defense overexpressed at 2 h, 48 h, and 72 h after inoculation with *Phytophthora cinnamomi*.

Transcript CC	Fold change			Best homology	CS orthologs	Reference	Notes
	2 h	48 h	72 h				
Innate Immune Response							
Transcript_55751	NA	3.22	2.91	PAMP-induced secreted peptide 2-like isoform X2	**Transcript_77746 (48 h)**	Hou et al. (2014)	Elicitor of immune response
Transcript_34749	NA	2.55	2.43	PAMP-induced secreted peptide 2-like isoform X2			
Calcium-binding proteins							
Transcript_33090	NA	9.62	NA	Calcium-binding EF-hand family protein	Transcript_73715	Zhang et al. (2014)	Involved in PTI and ETI
Transcript_31029	NA	2.29	NA	Probable calcium-binding protein CML44	NA		
Transcript_38648	NA	1.69	NA	Calcium-binding protein CP1	**Transcript_31927 (48 h)**		
Transcript_28157	NA	1.49	NA	Probable calcium-binding protein CML41	Transcript_34631 Transcript_41635 Transcript_30438		
Transcript_90554	NA	NA	4.79	Caltractin	**Transcript_49946 (72 h)**	Veeraraghavan et al. (2002)	Microtubule-organization center
Defense response–related transcription factors							
Transcript_31836	NA	7.07	NA	Ethylene-responsive transcription factor ERF098	NA	Lorenzo et al. (2003) Müller and Munné-Bosch (2015)	Possibly activated by ET/JA
**Transcript_40204**	NA	4.06	NA	Ethylene-responsive transcription factor ERF096-like	Transcript_29200		
**Transcript_28947**	NA	1.58	NA	Ethylene-responsive transcription factor 1B	Transcript_31232		
Transcript_13962	NA	1.4	NA	Ethylene-responsive transcription factor 1A	Transcript_37016		
Transcript_26888	NA	1.14	NA	Ethylene-responsive transcription factor 2	Transcript_37016		
Transcript_32820	NA	2.29	NA	Auxin-responsive protein IAA20-like	NA	Liscum and Reed (2002)	Repressors of early auxin response genes
**Transcript_38954**	NA	3.96	NA	Transcription factor MYB4-like	Transcript_20051	Park et al. (2010) Ambawat et al. (2013) Santos et al. (2017a)	Positive regulation of ROS (cold stress) Phenylpropanoid metabolism Probable candidate resistance gene to *P. cinnamomi*
Transcript_27881	NA	1.32	NA	Transcription factor MYB1-like	NA	Ambawat et al. (2013)	Phenylpropanoid metabolism
Transcript_27810	NA	1.79	NA	Myb-related protein 308-like	NA		
**Transcript_45220**	NA	2.97	NA	Transcription factor bHLH162-like	Transcript_50630	Goossens et al. (2017)	Possibly involved in JA signaling
Transcript_14479	NA	1.76	NA	Transcription factor bHLH93-like	NA		
Transcript_32413	NA	2.23	NA	Probable WRKY transcription factor 51	NA	Gao et al. (2011)	SA/JA regulation
Transcript_33178	NA	2.01	NA	Putative WRKY transcription factor 41	Transcript_47180 Transcript_29927	Higashi et al. (2008)	SA/JA regulation
**Transcript_29510**	NA	2.21	NA	NAC domain-containing protein 35-like	Transcript_36840	Yoo et al. (2007)	Cold tolerance
Transcript_55253	NA	1.11	NA	Sigma factor binding protein 1, chloroplastic	NA	Xie et al. (2010)	SA/JA regulation
Other defense response–related							
**Transcript_54590**	3.75	NA	NA	VQ motif-containing protein 1	Transcript_64448	Yuan et al. (2021)	Interacts with WRKY and MAPKs
Transcript_43018	NA	2.47	NA	MLO-like protein 11	Transcript_5695 Transcript_33460	Devoto et al. (2003)	May be involved in pathogen defense Regulated by Ca^2+^-dependent calmodulin binding
**Transcript_38690**	NA	2.2	NA	MLO-like protein 3	**Transcript_9042 (2 h)**	Kusch et al. (2019)	Possibly related to leaf senescence and SAR defense
Transcript_27434	NA	1.69	NA	Mitochondrial import inner membrane translocase subunit tim16-like	NA	Huang et al. (2013)	Protection from over-accumulation of ROS
**Transcript_38035**	NA	1.13	NA	dual specificity phosphatase Cdc25	Transcript_33351	Landrieu et al. (2004)	Positive regulation of CDKs
Transcript_19587	NA	2.2	NA	Pathogenesis-related protein 5	Transcript_37858	Ali et al. (2018)	Thaumatin-like proteins
Transcript_31660	NA	1.71	NA	Pathogenesis-related protein 5			
Transcript_43245	NA	4.17	NA	Pathogenesis-related protein 1-like	**Transcript_38325 (48 h)**		Antifungal
Transcript_43048	NA	3.41	4.02	Basic form of pathogenesis-related protein 1-like	**Transcript_40020 (2 h)**		
Transcript_44782	NA	3.52	3.94	Basic form of pathogenesis-related protein 1-like			
**Transcript_28346**	NA	1.3	NA	Thaumatin-like protein 1	Transcript_61853	Ali et al. (2018) Corredoira et al. (2012)	Promotes osmotic rupture in pathogen Candidate resistance gene to *P. cinnamomi*
Transcript_53769	NA	5.86	3.11	Thaumatin-like protein 1	NA		
Transcript_28076	NA	3.62	NA	Protein P21-like	**Transcript_35665 (48 h)**	Tachi et al. (2009)	Related to thaumatin-like protein PR-5 family
Transcript_29889	NA	3.2	NA	Protein P21-like			
Transcript_52224	NA	1.83	NA	Downy mildew resistance 6	Transcript_88453	Pavese et al. (2021)	Negative regulator of the SA pathway Candidate susceptibility gene
Anti-fungal							
Transcript_36960	NA	1.61	NA	Cysteine-rich repeat secretory protein 38	NA	Wang and Ng (2000) Sawano et al. (2007) Santos et al. (2017a)	*Cast_Gnk2-like* Anti-fungal secretory Candidate resistance gene
Transcript_51268	NA	1.05	NA	Stress-response A/B barrel domain-containing protein HS1	Transcript_43199 Transcript_37145	Park et al. (2007)	Heat stable protein with antifungal activity
Transcript_27096	NA	3.52	NA	Endochitinase EP3-like	NA	Corredoira et al. (2016) Serrazina et al. (2015)	Chitinase activity Probable candidate resistance gene to *Cryphonectria parasitica* and *P. cinnamomi*
Transcript_14022	NA	2.64	NA	Glycoside hydrolase	NA	Minic (2007)	Chitinase activity
Transcript_29138	NA	2.36	NA	Pathogenesis-related protein PR-4-like	NA	Ali et al. (2018)	Putative chitinase
**Transcript_19564**	NA	3.81	NA	Probable carboxylesterase 15	Transcript_45450	Marshall et al. (2003)	Serine hydrolase involved in plant-pathogen interactions
Transcript_14086	NA	1.97	NA	Glucan endo-1,3-beta-glucosidase, basic isoform-like	Transcript_61442	Ali et al. (2018)	PR-2 protein
Transcript_39159	NA	1.59	NA	Glucan endo-1,3-beta-D-glucosidase-like	NA		
Cell wall reinforcement/reorganization							
Transcript_93654	NA	6	NA	Pectinesterase 2	Transcript_83149	Santos et al. (2017a)	Apposition of pectates in the cell wall Candidate resistance gene to *P. cinnamomi*
Transcript_66245	NA	1.65	NA	Pectinesterase 2	Transcript_94639		
Transcript_44024	NA	1.8	NA	Probable xyloglucan endotransglucosylase/hydrolase protein 8	NA	Le Gall et al. (2015)	
Transcript_13832	NA	1.72	NA	Expansin-A20-like	NA	Bellincampi et al. (2014)	Plant cell wall loosening/extension
Transcript_32643	NA	2.28	2.61	Expansin-A4	**Transcript_67012 (2 h)**		
**Transcript_41406**	NA	2.2	NA	Cellulose synthase-like protein E1	Transcript_61318	Bellincampi et al. (2014) Santos et al. (2017b)	Cellulose synthesis Mapped on QTL related to *P. cinnamomi* resistance
Transcript_34502	NA	2.64	NA	Cellulose synthase-like protein E1	Transcript_81730		
Transcript_60659	NA	2.16	NA	Cellulose synthase-like protein E1			
Transcript_54206	NA	1.64	NA	Cellulose synthase-like protein E1			
**Transcript_32156**	NA	1.99	NA	Cellulose synthase-like protein G2	Transcript_80298		
Apoplast							
Transcript_20002	NA	4.11	NA	Dirigent protein 22-like	Transcript_45514 Transcript_69528	Paniagua et al. (2017)	Involved in lignin biosynthesis
**Transcript_30032**	NA	3.11	NA	Dirigent protein 19-like	Transcript_19911		
Transcript_27146	NA	2.17	NA	Dirigent protein 22-like	**Transcript_29539 (48 h)**		
**Transcript_27398**	NA	2.56	NA	Germin-like protein subfamily 2 member 1	Transcript_32389	Dunwell et al. (2008)	May play a role in pathogen defense
**Transcript_29211**	NA	2.08	NA	Germin-like protein subfamily 3 member 2	Transcript_29628		
Proteolysis							
Transcript_29062	NA	3.35	NA	Serine carboxypeptidase-like 18	NA	Liu et al. (2008)	May be related to oxidative stress response and increase of defense-related genes expression
**Transcript_70555**	NA	2.25	NA	Serine carboxypeptidase 1	Transcript_75953		
Transcript_14473	NA	NA	6.67	Serine carboxypeptidase-like 50 isoform X1	NA		
Transcript_76657	NA	NA	8.91	Cysteine proteinase 5	NA		
Transcript_42847	NA	1.59	NA	Subtilisin-like protease	NA	Figueiredo et al. (2014)	Induced after response to pathogen attack
Systemic Aquired Resistance							
Transcript_19822	NA	1.47	NA	EG45-like domain containing protein	NA	Ceccardi et al. (1998)	May counteract water and nutrient limitation
Phytohormone response and signaling pathways							
Transcript_14360	NA	1.88	NA	Auxin-responsive protein SAUR71-like	**Transcript_39048 (2 h; 48 h)**	Kant et al. (2009)	Negative regulation of auxin synthesis and transport
**Transcript_66563**	NA	1.8	NA	Auxin-responsive protein SAUR78	Transcript_28595		
Transcript_19889	NA	1.61	NA	Auxin-responsive protein SAUR78-like	**Transcript_31113 Transcript_32526 (2 h)**		
Transcript_43139	NA	1.49	NA	Auxin-responsive protein SAUR78-like			
Transcript_42379	NA	1.43	NA	Auxin-responsive protein SAUR32-like	NA		
**Transcript_57514**	NA	2.38	NA	Major allergen Pru ar 1-like	**Transcript_5581 (2 h)**	Tyurenkov et al. (2019)	Pathogenesis-related protein; binds to ABA
**Transcript_28416**	NA	2.19	NA	Protein C2-DOMAIN ABA-RELATED 11-like	Transcript_47764	Rodriguez et al. (2014)	ABA positive signaling
Transcript_29799	NA	2.27	NA	1-Aminocyclopropane-1-carboxylate oxidase	**Transcript_62058 (48 h)**	Qin et al. (2007)	Enzyme involved in the ethylene biosynthesis
Transcript_19675	NA	2.89	NA	Auxin efflux carrier component 5	NA	Adamowski and Friml (2015)	Auxin transporter in the ER
**Transcript_38601**	NA	1.84	NA	Salicylic acid-binding protein 2-like isoform X2	**Transcript_9109 (2 h)**	Tripathi et al. (2010)	SAR induction; converts MeSA into SA
Oxidative stress response							
Transcript_30258	NA	4.16	NA	Alpha-dioxygenase 1	NA	De León et al. (2002)	Promotes HR, local and SAR defense induced by SA
Transcript_32207	NA	2.18	NA	Hypothetical protein CMV_027614	Transcript_51858		
Transcript_30705	NA	1.22	NA	Glutaredoxin-C4	Transcript_32231	Cui et al. (2017)	ROS reduction Candidate resistance gene to *P. infestans*
Transcript_52457	NA	1.12	NA	Glutaredoxin-C4			
Transcript_26894	NA	2.62	NA	Peroxidase P7-like	NA	Ali et al. (2018) Welinder et al. (2002) Alcázar et al. (1995) Bhai et al. (2021)	PR-9 proteins Removal of H2O2 Involved in lignin biosynthesis Related to *Phytophthora* spp. defense
Transcript_60577	NA	2.46	NA	Peroxidase 5-like	Transcript_51858		
Transcript_39196	NA	2.37	NA	Peroxidase 5	**Transcript_50879 (2 h/48 h)**		
Transcript_38378	NA	3.3	1.75	Cationic peroxidase 1	Transcript_50014		
**Transcript_92430**	NA	6.33	NA	Protein DETOXIFICATION 16	Transcript_57057	Li et al. (2002)	Xenobiotic detox Involved in flavonoid metabolism
**Transcript_14144**	NA	2.12	NA	Protein DETOXIFICATION 33-like	Transcript_37612		
Transcript_13546	NA	1.61	NA	Protein detoxification 46, chloroplastic	Transcript_96414		
**Transcript_43626**	NA	1.11	NA	Protein DETOXIFICATION 35	Transcript_47670		
**Transcript_32432**	NA	2.57	NA	Superoxide dismutase [Mn], mitochondrial	Transcript_31080	Bowler et al. (1989)	Dismutation of superoxide radicals
**Transcript_28398**	NA	1.13	NA	Nucleoside diphosphate kinase 1	Transcript_65957	Fukamatsu et al. (2003)	ROS detoxification

Genes are divided into categories related to their Gene Ontology and putative functions. Best protein homology description (from blastx: cutoff e-value = 1E−5, HSP-hit coverage = 80%; or dammit: lowest e-value < 1E−05), and *Castanea sativa* (CS) ortholog transcripts are presented. Upregulated CS orthologs are in bold. Single-copy orthologs only upregulated by CC at the same time point are underlined and bold. Candidate resistance genes previously identified for *Castanea–P. cinnamomi* interaction are highlighted in blue. Genes probably related to susceptibility are highlighted in orange. Darker shades of green represent higher values of positive log2FC (fold change). NA, not applicable; PTI, pathogen-associated molecular pattern (PAMP) triggered immunity; ETI, effector-triggered immunity; ET, ethylene; SA, salicylic acid; JA, jasmonic acid; MeJA, methyl jasmonate; ABA, abscisic acid; ROS, reactive oxygen species; CDKs, cyclin-dependent kinases; MAPKs, mitogen-activated protein kinases; PR, pathogenesis-related; QTL, quantitative trait loci; SAR, systemic acquired resistance; ER, endoplasmic reticulum; HR, hypersensitive reaction.

*Castanea sativa* upregulated 28, 15, and 1 genes at 2 hai, 48 hai, and 72 hai, respectively. Only four genes were upregulated at more than one time point (2 hai and 48 hai) and putatively encode for an elicitor of immune response (PAMP-induced secreted peptide 2; Hou et al., 2014), an auxin repressor (Auxin-responsive protein IAA1; Liscum and Reed, 2002), a negative regulator of auxin synthesis and transport (auxin-responsive protein SAUR71-like; Kant et al., 2009), and a protease with several roles in biotic stress response such as removal of hydrogen peroxide (H_2_O_2_) and lignin polymerization (peroxidase 5; Welinder et al., 2002). At 2 hai, *C. sativa* is mainly upregulating transcription factors known to mediate gene expression related to biotic stress responses, such as ethylene-response factors (*ERF*s; Lorenzo et al., 2003; Müller and Munné-Bosch, 2015), *WRKY*s (Eulgem et al., 2000), and *protein radialis-like 3* (MYB-related; Yanhui et al., 2006). It also upregulates genes involved in phytohormone response and signaling pathways (mostly negative regulators of auxin synthesis and transport), oxidative stress response, and cell wall reinforcement or reorganization. A calcium-dependent NADPH oxidase that generates superoxide—*respiratory burst oxidase homolog protein B* (*RBOHB*; Kobayashi et al., 2007)—was also upregulated at 2 hai. The genes upregulated at 48 hai are anti-fungal (*pathogenesis-related protein 1-like*; Ali et al., 2018; *Acidic endochitinase Q*; Payne et al., 1990; and *thaumatin-like protein*, Corredoira et al., 2012), are probably involved in lignin synthesis (*putative laccase-9*; Turlapati et al., 2011; and *dirigent protein 22-like*; Paniagua et al., 2017), and encode Ca^2+^-binding proteins. The gene with the highest fold change (8.37) was the protease *Cysteine proteinase 3*, the only gene upregulated at 72 hai. From the 41 genes, only 11 had orthologs in *C. crenata* also being upregulated (**Table 1**).

*Castanea crenata* only upregulated *VQ motif-containing protein 1* (interacts with WRKY and MAPKs; Yuan et al., 2021) at 2 hai, followed by a transcriptional burst at 48 hai of 86 genes from all categories (**Table 2**). At 72 hai, the number of upregulated genes decreased to 10 which included two elicitors of immune response (*PAMP-induced secreted peptide 2-like isoform X2*; Hou et al., 2014), one component of microtubule-organization center (*Caltractin*; Veeraraghavan et al., 2002), three anti-fungal (*basic form of pathogenesis-related protein 1-like*; *thaumatin-like protein 1*; Ali et al., 2018), one cell wall loosener (*expansin-A4*; Bellincampi et al., 2014), two proteases (*Cysteine proteinase 5* and *serine carboxypeptidase-like 50 isoform X1*; Balakireva and Zamyatnin, 2018), and one oxidative stress reducer (*Cationic peroxidase 1*; Welinder et al., 2002). Several genes with distinct functions in the defense process (e.g., cell wall reinforcement, proteolysis, and oxidative stress response) had a fold change higher than 5 (highlighted in darker green in **Table 2**). From the total 90 genes, 15 had orthologs in *C. sativa* being upregulated (**Table 2**).

### 3.8 *Castanea crenata* upregulated several transcripts involved in phenolic compounds’ metabolic pathways

The KEGG metabolic pathways analysis identified 20 and 71 pathways that were assigned to 19 and 81 DEGs of *Castanea sativa* and *Castanea crenata*, respectively (**Supplementary Tables 21**, **22**). Phenolic compounds are important for early resistant defense responses against *Phytophthora cinnamomi* (Fernandes et al., 2021). Metabolic pathways related to the biosynthesis of phenolic compounds were assigned to several *C. crenata* upregulated DEGs at 48 hai: 1) flavonoid biosynthesis [five upregulated transcripts; e.g., *chalcone isomerase* and *anthocyanidin reductase ((2S)-flavan-3-ol-forming)*]; 2) flavone and flavonol biosynthesis (one upregulated transcript, *cytochrome P450 CYP736A12-like*); 3) phenylpropanoid biosynthesis (one upregulated transcript, *caffeoyl-CoA O-methyltransferase*); 4) phenylalanine, tyrosine, and tryptophan biosynthesis (six upregulated transcripts; e.g., *L-aspartate oxidase*, *chloroplastic* and *Bifunctional 3-dehydroquinate dehydratase/shikimate dehydrogenase*, *chloroplastic*); and 5) rerpenoid backbone biosynthesis (2 upregulated transcripts; *putative dihydroflavonol 4-reductase* and *geranylgeranyl pyrophosphate synthase*, *chloroplastic-like*; **Supplementary Tables 12**, **22**). No phenolic compound pathway was assigned to *C. sativa* DEGs (**Supplementary Table 21**).

## 4 Discussion

Dual-transcriptomics can capture both host and pathogen transcriptomes (Westermann et al., 2012) when it is unfeasible to separate the interacting organisms. Studying the associated gene expression changes simultaneously has allowed a more comprehensive understanding of the dynamics of several plant-pathogen interactions (Hayden et al., 2014; Meyer et al., 2016; Evangelisti et al., 2017; Liao et al., 2019).

### 4.1 *Phytophthora cinnamomi* during chestnut infection

#### 4.1.1 PAMPs and effectors putatively linked to chestnut defense response

The recently available reference genome of *Phytophthora cinnamomi* (Engelbrecht et al., 2021) represents a valuable resource that provides insight into pathogenicity determinants in this species, as previously demonstrated for other *Phytophthora* species, such as *P. infestans*, *P. sojae*, and *P. ramorum* [reviewed by Jiang and Tyler (2012)]. Several oomycete PAMPs were expressed by *P. cinnamomi* in the roots of susceptible and resistant chestnuts. Secretory OPEL protein is present in the cell wall of *Phytophthora* spp. and can induce the expression of several SA-responsive genes (e.g., *PR1* and *PR5*) and callose deposition (Chang et al., 2015). These host responses were detected in *Castanea crenata* and *Castanea sativa*, which may indicate that both species could recognize Secretory OPEL protein. This protein can also induce cell death and ROS accumulation (Chang et al., 2015). *OPEL* was more expressed in *C. crenata* than in *C. sativa* at 2 hai and was not DE between 72 hai and 48 hai in either chestnut species. This may indicate that this PAMP is more expressed during penetration and establishment of biotrophy in this interaction. Indeed, *OPEL* is highly expressed by *P. parasitica* after plant inoculation, reaching its peak at 24 h and declining at 48 hai and 72 hai (Chang et al., 2015). *Transglutaminase elicitor M81C* was also more expressed in the resistant chestnut at 2 hai. M81C displays elicitor-like activity in *P. infestans* due to the presence of a 100% conserved region of 13 amino acids (Pep-13) initially identified as a PAMP in *P. sojae* (Nürnberger et al., 1994; Fabritius and Judelson, 2003). Pep-13 could be responsible for a quick increase in cytosolic Ca^2+^ concentration, as previously reported to occur in parsley (Blume et al., 2000). The putative early perception of OPEL and M81C by *C. crenata* may be inducing HR and callose deposition observed after *P. cinnamomi* penetration and biotrophy establishment (Fernandes et al., 2021).

*Phytophthora* spp. are an example of a pathogenic and evolutionary success because they quickly evolved diverse effector gene complements. RxLR proteins [including Avirulence (Avr) proteins] and CRN are cytoplasmic effectors that are targeted to the plant nucleus and suppress host defenses, promoting disease in susceptible plants. However, only RxLRs are currently known to activate avirulence activity in resistant plants [reviewed by Naveed et al. (2020)]. In *C. sativa*, *RxLR-like* genes were upregulated at 48 compared to 72 hai, as the rest of the effectors of pathogen attack, possibly contributing to the susceptibility of the host as indicated by the enriched GO *modulation by symbiont of host defense-related programmed cell death*. CRNs can cause crinkling and necrosis (Hardham and Blackman, 2018), two symptoms associated with *P. cinnamomi–*induced root rot in chestnuts (Santos et al., 2015a). Suppressor of necrosis 1 can also be translocated to the plant nucleus and suppress the induction of programmed cell death, as previously reported during the biotrophy of tomato–*P. infestans* interaction (Kelley et al., 2010). Interestingly, *putative suppressor of necrosis 1* is more expressed in *C. crenata* roots at 2 hai and 72 hai than that in *C. sativa*. Still, HR-related cell death was observed in the resistant chestnut (Fernandes et al., 2021).

#### 4.1.2 Nutrient uptake during chestnut infection

The apoplast plays an important role in nutrient uptake and signal exchange, and it is where the initial interactions between plants and pathogens occur (Aung et al., 2018). The apoplastic effector aldose 1-epimerase (AEP1) is an oomycete PAMP and also a virulence factor that mediates *P. sojae* extracellular cellular sugar uptake (Xu et al., 2021). Hemibiotrophic oomycetes need a biotrophy phase to acquire nutrients from the host living cells before switching to a necrotrophic lifestyle in which nutrients are obtained from killing host cells. Indeed, the allocation of energy-rich compounds is very important during the biotrophic growth of *P. cinnamomi* (Oßwald et al., 2014) and other *Phytophthora* spp (Xu et al., 2021). Results suggest that transporters potentially involved in *Phytophthora* nutrition are more expressed by *P. cinnamomi* in both chestnut species at 48 hai. It is likely that the pathogen was uptaking nutrients to increase growth and colonize roots. There are limited studies on oomycete nutrition; however, it has been previously reported that *Phytophthora* spp. can assimilate nutrients from their plant host and have metabolic switches during host infection (Rodenburg et al., 2019).

#### 4.1.3 Biotrophy to necrotrophy switch

After 3 to 6 days, *Phytophthora* spp. biotrophy is followed by a necrotrophic phase when necrotic lesions appear, and sporangia develop (Van West et al., 1998; Maia et al., 2012; Crone et al., 2013; Redondo et al., 2015; Ah-Fong et al., 2017). By 72 hai, *P. cinnamomi* developed resistance structures (chlamydospores) and switched to necrotrophic growth in *C. sativa*’s roots. Elicitins are oomycete PAMPs that, when not recognized by the host, induce structural changes in plant cells and participate in sterol scavenging, helping with pathogen growth and disease development (Hardham and Blackman, 2018). Knocking out the expression of elicitins resulted in reduced pathogen penetration and colonization in cork oak and European chestnut (Horta et al., 2010; Maia et al., 2012). Data suggest that genes encoding for elicitins/elicitins-like are mostly expressed in *C. sativa* at 48 hai and 72 hai. *Necrosis-inducing like protein* (*NLP*) genes were also found mainly upregulated in *C. sativa* at 48 hai and can act in the host cytoplasm as toxins, causing plant necrosis by plasma membrane destruction and cytolysis (Oßwald et al., 2014; Hardham and Blackman, 2018). In previous reports, the expression of NLP *npp1* (*necrosis-inducing Phytophthora protein 1*) in *C. sativa* radicles increased at 36 hai (Martins et al., 2019). NLPs can be recognized during PTI and ETI [reviewed by Naveed et al. (2020)] and have been related to the switch to necrotrophy and acceleration of host cell death during this stage of the pathogen’s growth in *Phytophthora* spp (Qutob et al., 2002; Jupe et al., 2013).

#### 4.1.4 Sporulation-related genes

The successful colonization of an oomycete during a compatible host interaction usually ends in sporulation, which has been previously detected a few days after infection (e.g., 72 hai and 96 hai) in plant–*Phytophthora* spp. interactions (Hardham, 2007; Judelson et al., 2009; Jupe et al., 2013). *DEAD/DEAH box RNA helicase* and *Zinc finger*, *C2H2*, two genes related to sporulation in *Phytophthora* spp. (Walker et al., 2008; Wang et al., 2009) were found more expressed by *P. cinnamomi* in the roots of the susceptible chestnut. During host infection, *P. infestans* starts upregulating sporulation-related genes pre-sporulation (48 hai and 72 hai) and reaches a peak post-sporulation (96 hai; Judelson et al., 2009). Sporangium development was not observed in infected chestnuts in this work or in the work of Fernandes et al. (2021); however, future studies should be developed to verify if and when sporulation is achieved in chestnut species.

### 4.2 Chestnut transcriptomes during *Phytophthora cinnamomi* infection

By using the *Phytophthora cinnamomi* genome, reads of the pathogen were separated from the host, and the development of single *de novo* assemblies for the two hosts was possible. After filtering *de novo* transcriptomes for Streptophyta, the number of remaining transcripts in both species was higher than the previously reported transcriptomes of chestnut roots inoculated with this pathogen (approximately 14,000 vs. 8,000 transcripts; Serrazina et al., 2015). The number of DEGs was also higher (488 vs. 305 in *Castanea sativa*; 1,556 vs. 283 in *Castanea crenata*; Serrazina et al., 2015). This was expected because the 2015 transcriptomes were developed with 454 sequencing, which typically results in lower-depth datasets when compared to Illumina (Ward et al., 2012). In a general analysis, the results reported in this work suggest similar defense responses as previously reported (Serrazina et al., 2015), such as recognition of the pathogen by both species and upregulation of genes related to HR, SA, and ET/JA-dependent signaling pathways, antifungal compounds and enzymes, and cell wall reinforcement.

#### 4.2.1 Chestnut’s recognition of *Phytophthora* cinnamomi

Plants recognize *Phytophthora* spp. by sensing several elicitors [reviewed by Naveed et al. (2020)]. Although this knowledge is still scarce, a few *Phytophthora* PAMPs that can induce PTI have been identified, and plant perception models have been proposed [reviewed by Naveed et al. (2020)]. The recognition of some *Phytophthora* PAMPs is done by a PRR that associates with the LRR RLK BAK1 (BRI1-associated kinase-1), a known hub in defense responses (Heese et al., 2007). The recognition of elicitins, NLPs [reviewed by Naveed et al. (2020)], and AEP1 (Xu et al., 2021) is BAK1-dependent. So far, there are only two BAK1 cognate PRRs known to recognize *Phytophthora* PAMPs, the Elicitin response receptor (ELR) and RLP23, which recognize the INF1 elicitin and nlp20, respectively [reviewed by Naveed et al. (2020)]. However, these were not identified in this work. *BAK1* was upregulated by *C. crenata* at 48 hai, suggesting PRR-BAK1 association as a part of the Japanese chestnut machinery to perceive *P. cinnamomi*’s PAMPs. *Castanea crenata* also upregulates several genes encoding putative PRRs that might interact with BAK1 during pathogen perception and PTI induction. *Castanea sativa* had no *BAK1* ortholog detected in the transcriptome and few putative PRR genes DE during infection. A higher number of PRRs was expected in *C. crenata* since it co-evolved with *P. cinnamomi* (Crandall et al., 1945). The selection pressure ultimately led to the evolution of PRRs to recognize PAMPs/effectors as danger signals. A gene putatively encoding the lysin motif RLK (LysM-RLK) LYK5 is also overexpressed by *C. crenata* at 48 hai. In *Arabidopsis*, LYK5 receptor forms a chitin-inducible complex with CERK1 to induce host immunity (Cao et al., 2014). Cheng et al. (2019) proposed that chitin may act as a PAMP in *Phytophthora* spp. and is recognized by LYK5-CERK1. Although the presence of chitin is yet to be reported in *Phytophthora* spp., chitin synthases (enzymes responsible for chitin biosynthesis) are present in their genomes and expressed during some developmental stages and after infection (Cheng et al., 2019). It has also been recently shown that CERK1 can bind to synthetic β-1,3-glucan oligosaccharides (components of oomycete cell walls; Wang et al., 2018). Since CERK1 and LYK5 are very similar receptors (Cao et al., 2014), they might share the capability of attaching to β-1,3-glucan. Nevertheless, if *C. crenata* LYK5 were able to recognize chitin or β-1,3-glucan, then its signaling would have to be transduced downstream by associating with another receptor since its kinase domain is inactive (Cao et al., 2014).

#### 4.2.2 *Castanea sativa* defense response to the pathogen

The earliest host events reported after receptor-mediated perception of *Phytophthora* PAMPs involve Ca^2+^ influx (within 30 s after PAMP perception) and ROS bursts (within minutes). Also, calcium, MAPK, and hormonal signaling (JA, SA, and ET) pathways are activated, leading to transcriptional reprogramming and activation of PTI defenses that include callose deposition, HR, expression of defense genes, accumulation of antimicrobials, and SAR [reviewed by Naveed et al. (2020)]. *Castanea sativa* seems to have a smaller but quicker transcriptional change in response to *P. cinnamomi* than *C. crenata.* At 2 hai, the susceptible chestnut upregulates several genes related to resistance responses such as pathogen-induced ROS burst (*RBOHB*; Kobayashi et al., 2007), ET/JA (*ERF*s; Lorenzo et al., 2003; Müller and Munné-Bosch, 2015) and SA signaling pathways [*salicylic acid-binding protein 2-like* (*SABP2-like*); Tripathi et al., 2010]. However, the decrease of upregulated defense-related genes throughout the time points suggests a decline in the response, unlike the delayed response to the pathogen previously proposed by Santos et al. (2017a). At 48 hai, *C. sativa* continued to upregulate several transcripts encoding for proteins putatively involved in *Phytophthora* spp. defense, such as metalloendoproteinase 2-mmp, peroxidase 5, and thaumatin-like. *Metalloendoproteinase 2-mmp* leads to the production of antimicrobial peptides and is highly induced in soybean after *Phytophthora* infection (Liu et al., 2001); peroxidase activity was suggested as a marker for *Phytophthora* resistance in Black pepper (Bhai et al., 2021), and thaumatin-like may induce osmotic rupture in the pathogen and it is proposed as a chestnut candidate resistance gene to *P. cinnamomi* (Corredoira et al., 2012). *PAMP-induced secreted peptide 2* (*PIP2*, elicitors of immune response in *Arabidopsis*; Hou et al., 2014), MAPKs, and several calcium-binding proteins are also upregulated at 48 hai, which could be players on PTI-induced defenses. These results raise the hypothesis that *P. cinnamomi* may not be able to suppress the classic host defense pathways induced by the susceptible chestnut during the early stages of infection. However, *C. sativa* transcriptome suggests a lack of defenses when the oomycete begins necrotrophic growth. Similar results have been previously reported by Vargas et al. (2012) for the interaction of maize with the hemibiotroph *Colletotricum graminicola*. In this interaction, *C. graminicola* is exposed to detrimental conditions during the biotrophic phase due to the host immune system responses (e.g., ROS burst). The switch to necrotrophy is interpreted as a mechanism to avoid contact with the defense molecules produced by the host’s living cells and, therefore, achieve full pathogenicity (Vargas et al., 2012). Nevertheless, other factors may be contributing to the European chestnut’s defense decrease such as the overexpression by 2.4 fold change of *BAP2* at 48 hai. This gene’s product might be suppressing attempts of programmed cell death induced by *C. sativa* after pathogen attack, a function demonstrated with *Arabidopsis BAP2* loss of function mutants (Yang et al., 2007).

#### 4.2.3 *Castanea crenata* initial defense

*Castanea crenata* only upregulated one gene at 2 hai by 3.75 fold change, *VQ motif-containing protein 1*. This gene’s product may be interacting with WRKY transcription factors and MAPK signaling cascades (Yuan et al., 2021), two well-known participants of the plant immune system (Meng and Zhang, 2013; Wani et al., 2021). Indeed, VQ motif-containing proteins were suggested to control plant response to *P. parasitica* by having their transcripts overexpressed immediately after pathogen penetration, enabling the host to cope with effector-induced cellular reprogramming (Le Berre et al., 2017). The low number of DEGs at 2 hai may indicate that *C. crenata*’s constitutive levels of gene expression could be sufficient as a first defense after pathogen penetration. Indeed, at this time point, the Japanese chestnut presented callose deposition around intracellular hyphae, accumulation of phenolic compounds on the cell wall, and HR-related cell death (Fernandes et al., 2021). This was hypothesized by Santos et al. (2017a) after verifying in non-inoculated Japanese chestnuts, high expression levels of several genes related to resistance responses such as pathogen recognition (*Cast_LRR-RLK*), transcription factors (*Cast_Myb4*), antifungal activity (*Cast_Gnk2-like*), and cell wall strengthening (*Cast_PE-2*). These genes were also found to be upregulated at 48 hai (Santos et al., 2017a), as reported in this work.

#### 4.2.4 Antifungal activity

*Cysteine-rich repeat secretory protein 38* (*Cast_Gnk2-like*) encodes for an antifungal secretory protein first identified in *Ginkgo biloba* seeds, also known as Ginkgobilobin-2 (Wang and Ng, 2000; Sawano et al., 2007; Santos et al., 2017a). This protein may also activate actin-dependent cell death (Gao et al., 2016). *Cast_Gnk2-like* was linked to the constitutive defense of *C. crenata* due to putatively preventing the pathogen’s growth through chemical properties or by inducing HR-like cell death (Santos et al., 2017a). This gene was upregulated in *C. crenata* in this work, and no ortholog was identified in the susceptible species transcriptome. This is probably due to the extremely low expression of this gene described by Santos et al. (2017a). The functional validation of *Cast_Gnk2-like* as a resistance gene is ongoing through genetic transformation of susceptible species (McGuigan et al., 2020; Serrazina et al., 2022) and the study of the putative anti-oomycete activity of the encoded protein (Colavolpe et al., 2023).

Genes encoding for proteins with antifungal activity (basic form of PR protein 1-like; thaumatin-like protein 1; Corredoira et al., 2012; Ali et al., 2018) were still highly upregulated (3 to 4 fold change) at 72 hai in *C. crenata.* Also, at this time point, *P. cinnamomi* is expressing a *Catalase* in *C. crenata* 2.22 fold more than in *C. sativa*, which may indicate the presence of ROS. Catalases are suggested to act as *Phytophthora* effectors against plant defenses by breaking down H_2_O_2_ into water and oxygen (Hardham and Blackman, 2018).

#### 4.2.5 Phenylpropanoid pathway and cell wall reinforcement

Lignin and phenylpropanoid biosynthesis involvement is well-known during plant defense against biotic stresses (Miedes et al., 2014). *Castanea crenata* had a considerably higher upregulation of genes related to the biosynthesis of these compounds compared to *C. sativa.* The resistant chestnut overexpressed several genes encoding for enzymes and MYB transcription factors involved in the phenylpropanoid metabolism and several dirigent proteins involved in lignin biosynthesis (Paniagua et al., 2017). The accumulation of phenolic compounds in the cell walls is detected 30 min after inoculation in *C. crenata* and 72 hai in *C. sativa* (Fernandes et al., 2021). The buildup of phenolic compounds leads to lignin polymerization (Pomar et al., 2002), which has been shown to be an active defense against *P. cinnamomi* in later stages of the infection in *Eucalyptus* spp (Cahill and McComb, 1992), avocado (van den Berg et al., 2018a), and chestnut Euro-Asian hybrids [P. Fernandes and M. C. Silva, *Unpublished data*, as cited in the work of Fernandes et al. (2021)]. Two *pectinesterase 2* and five *cellulose synthase-like* were upregulated at 48 hai by *C. crenata* and are putative resistance genes to *P. cinnamomi* acting on cell wall reinforcement (Santos et al., 2017a, b). Interestingly, mutating a cellulose synthase gene in *Arabidopsis* reduced the cellulose, stimulating the production of lignin and consequently increasing the resistance to powdery mildew pathogens [reviewed by Bellincampi et al. (2014)].

The accumulation of phenolic compounds reinforces *C. crenata* cell walls and blocks of *P. cinnamomi* hyphae, which were occasionally found dead at 72 hai (Fernandes et al., 2021). The Japanese chestnut cells possibly induced defenses that created a hostile environment for the oomycete, such as phenolic compound accumulation, antimicrobial compounds, and ROS. Many phenolic compounds from chestnuts may exhibit antimicrobial activity (Silva et al., 2020).

#### 4.2.6 Microtubule and cytoskeleton organization

Reorganization of the cytoskeleton and architecture changes can be players in the plant immune system (Li and Staiger, 2018). It has been previously shown that plants are able to focus actin microfilaments, aggregate ER membrane, and accumulate Golgi bodies near oomycete penetration sites (Takemoto et al., 2003), suggesting a rapid relocation of defenses. Actin filament organization is an enriched GO term at 48 hai in *C. crenata*, which may suggest relocation of defenses after *P. cinnamomi* infection. Several other GO terms related to cytoskeleton and microtubule processes are enriched at 72 hai. However, Takemoto et al. (2003) did not find microtubules focused on the penetration site suggesting a different function for the genes related to these GO terms during the late stages of *P. cinnamomi* infection. Indeed, microtubule and cytoskeleton organization have been previously related to HR cell death in plants infected with the pathogen cowpea rust fungus (Škalamera and Heath, 1998). The same authors also suggested that microtubules are involved in the deposition of phenolics by adjacent living cells. Both HR and phenolic compound deposition were observed in *C. crenata* cells infected with *P. cinnamomi* (Fernandes et al., 2021). However, cytoskeleton dynamics, namely, actin and microtubule changes, should be addressed microscopically to study these hypotheses.

#### 4.2.7 SA, JA, and ET signaling

Intersignaling between the stress-induced hormones SA, JA, and ET is considered the core of immunity, and its importance has been established for a long time (Pieterse et al., 2012). In previous reports, specific roles have been given to each hormone pathway during pathogen attacks. The SA pathway is associated with the response against several biotrophic and hemibiotrophic pathogens. ET/JA pathway seems to be recognized as the main defense against necrotrophic pathogens, wounding, and herbivores. However, studies with *P. cinnamomi* show the complex interplay between these phytohormones. So far, no predominant pathway is responsible for restraining this pathogen’s growth [reviewed by van den Berg et al. (2021)]. Actually, signature genes related to SA (e.g., *pathogenesis-related 1-like* and *pathogenesis-related 5*; Ali et al., 2018) and to JA (e.g., *pathogenesis-related 4*; Ali et al., 2018) signaling pathways were upregulated at the same time in *C. crenata*, along with transcription factors that might have been participating in SA/JA regulation (e.g., *WRKY51*; Gao et al., 2011; and *WRKY41*; Higashi et al., 2008). *ERF*s are activated by ethylene and jasmonate (Lorenzo et al., 2003) and have been shown to confer resistance to *Phytophthora* spp. by participating in the reprogramming of gene expression (Yu et al., 2020) and upregulation of PR genes in overexpressing mutants (Zhao et al., 2017). *ERF*s were considered involved in the regulation of defense responses in chestnuts by Serrazina et al. (2015).

#### 4.2.8 Auxin signaling

Understanding the role of growth-promoting hormones (auxins, cytokinins, gibberellic acid, and abscisic acid) in homeostasis regulation during plant-pathogen interactions is far behind when compared to SA, ET, and JA. The importance of auxin signaling pathways during plant–*Phytophthora* interactions has been previously proposed (Evangelisti et al., 2013; Eshraghi et al., 2014). The present results suggest a response to auxin and downregulation of auxin-signaling pathways at 48 hai in the resistant chestnut. In addition to the GO-enriched terms in downregulated genes (*auxin-activated signaling pathway; auxin transport*), *C. crenata* is overexpressing several transcripts that are rapidly and transiently induced in response to auxins such as the *Aux/IAA* (*auxin-responsive protein IAA20-like*) and *Small auxin-up RNA*s (*SAUR*s; Hagen and Guilfoyle, 2002). Aux/IAA proteins are short-lived transcription factors that repress auxin early responses (Liscum and Reed, 2002). The function of the SAUR family is not very well known; however, some members have been proposed as negative regulators for auxin synthesis and transport (Kant et al., 2009) and may play a role in the recovery or control of stress response levels caused by auxin (Markakis et al., 2013). A similar overexpression seems to be happening in the susceptible species but with fewer genes and earlier in the infection process (2 hai/48 hai). *Castanea crenata* was also overexpressing *PIN5* (*auxin efflux carrier component 5*), frequently localized in the endoplasmic reticulum and likely mediating auxin transport into this cellular compartment (Mravec et al., 2009). PIN5 appears to be necessary for fine-tuning auxin function and is most likely regulating the hormone’s homeostasis by subcellular auxin compartmentalization in the ER lumen (Mravec et al., 2009). Contrary, *C. sativa* is overexpressing *PIN4* (*auxin efflux carrier component 4*), which plays a role in creating a sink for auxin in the columella cells (Adamowski and Friml, 2015). The interpretation of these results is not clear; however, it may suggest a temporary decrease of root growth as a trade-off between defense and a resistance response in *C. crenata* to avoid *P. cinnamomi* manipulation of this phytohormone pathway as reported for *Arabidopsis*–*P. parasitica* (Evangelisti et al., 2013). During penetration *P. parasitica* transiently upregulates the RXLR effector PSE1, which is suggested to modulate auxin content in the root by inducing the host to overexpress PIN4 and PIN7 transporters, leading to an accumulation of auxin in the columella. This may facilitate pathogen penetration and suppress plant cell death (Evangelisti et al., 2013).

#### 4.2.9 Protein degradation

The 26S regulatory subunit plays a central role in the degradation of plant proteins through the ubiquitin/26S proteasome system and can be responsible for the degradation of several substrates involved in hormone signaling and plant defense (Dreher and Callis, 2007; Dielen et al., 2010). Some subunits may also inhibit pathogen effectors and trigger PAMP and ETI responses (Park et al., 2012; Üstün and Börnke, 2014). Given its important regulatory role in plant defense, it is a target for utilization by pathogens such as *P. infestans*, which inhibits plant immunity by modulating proteasome activity (Bos et al., 2010). 26S proteasome subunits have been previously related to host resistance to *P. cinnamomi* after observation of increased susceptibility in *Arabidopsis* defective mutants (Eshraghi et al., 2014). It was also related to soybean-enhanced resistance to *P. sojae*, where overexpressing a 26S proteasome subunit gene improved the activity of antioxidant enzymes and decreased ROS. In contrast, knockdowns increased ROS accumulation and inhibition of antioxidant enzymes (Liu et al., 2021). The high fold change of a 26S proteasome subunit gene in *C. crenata* and several proteases may indicate an important role in protein degradation during *P. cinnamomi* infection. Indeed, this pathogen overexpresses in *C. crenata* tissues (compared to *C. sativa*) a *Kazal-like serine protease inhibitor*, a protease inhibitor usually expressed as a defense against host responses (Hardham and Blackman, 2018).

#### 4.2.10 Similarity to chestnut blight defense

Another serious threat to chestnuts is chestnut blight, which is caused by the fungi *Cryphonectria parasitica* (Murr.) Barr. Candidate resistance genes for *C. parasitica* obtained from studies with *C. mollissima* (Cm) and *C. seguinii* (Cse) are reviewed by Nelson et al. (2014). Several of these genes are similar to the ones in this work including *Laccase-like protein/diphenol oxidase* (Cm), *Ethylene Transcription Factor* (Cm), *Thaumatin-like protein* (Cm), *Caffeoyl-CoA-O-methyltransferase* (Cm), *Peroxidase* (Cm), *Subtilisin-like protease* (Cse), and *Glucan endo-1,3-glucosidase* (Cm) (Nelson et al., 2014). Indeed, Serrazina et al. (2015) suggested that responses of European and Japanese chestnuts to *P. cinnamomi*, and American and Chinese chestnuts to *C. parasitica* were similar. The DEGs after pathogen attack were involved in common defenses such as pathogen recognition, JA signaling, gene regulation by MYB, and ethylene-responsive transcription factors, HR and cell lignification, and anti-fungal proteins (Barakat et al., 2009, 2012; Serrazina et al., 2015).

### 4.3 Conclusions

The generalized lack of symptomology and high survival in Asian chestnut species and hybrids resistant to *Phytophthora cinnamomi* is far from being unraveled. However, this work reveals new and valuable contributions to help enlighten these interactions.

*Phytophthora cinnamomi* had a higher expression of different effectors in resistant and susceptible chestnuts. *RxLRs* and *CRLs* were more expressed in *Castanea crenata* and *elicitins* and *NLPs* in *Castanea sativa.* Considering the low number of potential PRR genes overexpressed in *C. sativa* after infection and the undetected *BAK1*, it is likely that elicitins and NLPs have a significant role in the cellular decline of this chestnut species. Additionally, *P. cinnamomi*’s switch to necrotrophy in the susceptible chestnut might be associated with the evasion of host responses from living cells followed by a pathogenicity increase. Genes related to sporulation were only overexpressed in *C. sativa*, whereas genes related to pathogen defense against host responses are more expressed in *C. crenata.*

The hypothesis of *P. cinnamomi* PAMPs perception and PTI activation via PRR-BAK1 should be explored. The main difference between non-resistant and resistant host response in chestnut–*P. cinnamomi* interactions might be more quantitative than qualitative, as previously suggested by Serrazina et al. (2015) and Zhebentyayeva et al. (2019). This has been recorded in other plant–*Phytophthora* interactions (Vleeshouwers et al., 2000) and chestnut-*Cryphonectria* interactions (Huang, 1996; Kubisiak et al., 1997, 2013). *Castanea sativa* is able to recognize *P. cinnamomi* and upregulate genes involved in defense responses, which include at 2 hai: ROS burst, regulation of defense genes by WRKY and possibly MAPKs (through VQ1), ET/JA signaling regulation by ERFs, SA signaling regulation by SABP2-like, negative regulation of auxin by SAUR-like, proteases, and oxidative stress response; at 48 hai: possibly Ca^2+^ signaling and MAPK signaling cascades, antifungal proteins, lignin metabolism, and proteases; and at 72 hai: most defenses are possibly disabled. However, the number of genes upregulated to induce a specific response was much lower than the induced by the resistant chestnut. Therefore, the defenses induced by *C. sativa* after *P. cinnamomi* attack are not enough to restrain the pathogen’s colonization, which is able to start the necrotrophic growth at 72 hai. The pathogen possibly manipulates *C. sativa* by translocation of effectors to the plant nucleus, which, together with the host expression of *BAP2*, might halt the induction of programmed cell death and several other defense mechanisms. The role of *BAP2* as a putative suppressor of PCD in chestnuts should be considered for further studies.

The resistant chestnut possibly has constitutive levels of gene expression that do not require upregulation of several genes for a first defense response. *Castanea crenata* probably recognizes most of the pathogen PAMPs/effectors, considering the high number of PRR genes upregulated, and induces PTI-, ETI-, and SAR-related defense responses due to a transcriptional burst at 48 hai. This includes Ca^2+^ and MAPK signaling cascades, HR-cell death, regulation of ET/JA and SA signaling pathways, repression of auxin, phenolic compound accumulation, cell wall reinforcement, antifungal proteins, proteases, and oxidative stress response. At 72 hai, it maintains some active defenses that continue to restrain the pathogen and, consequently, reduce the area of infection.

The outcomes of this work reveal new information about chestnut–*P. cinnamomi* interaction and indicate several new genes as potential targets for functional validation, such as several PRRs from *C. crenata*, susceptibility genes from *C. sativa*, and pathogen effectors. These genes could inform ongoing programs to improve the resistance of susceptible chestnuts by providing new baselines for the development of molecular markers (fast genomic selection), and host and pathogen genes for genetic transformation (overexpression/knockout; induced gene silencing by RNA interference). These results will also be useful in understanding the interaction of *P. cinnamomi* with other plant species, mainly woody species belonging to the Fagaceae family.

## Data Availability

The datasets generated in this study have been deposited in the NCBI BioProject database (https://www.ncbi.nlm.nih.gov/bioproject/), BioProject accession number PRJNA1108966.
