# Supplementary material for: Dual transcriptomic analysis reveals early induced Castanea defense-related genes and Phytophthora cinnamomi effectors
Source: Front Plant Sci. 2024 Aug 12;15:1439380. doi: 10.3389/fpls.2024.1439380 (PMC11345161; doi:10.3389/fpls.2024.1439380)
Supplement: Supplementary file 1 [file DataSheet_1.zip › Data sheet 1/Supplementary figures.docx]

Supplementary Figures


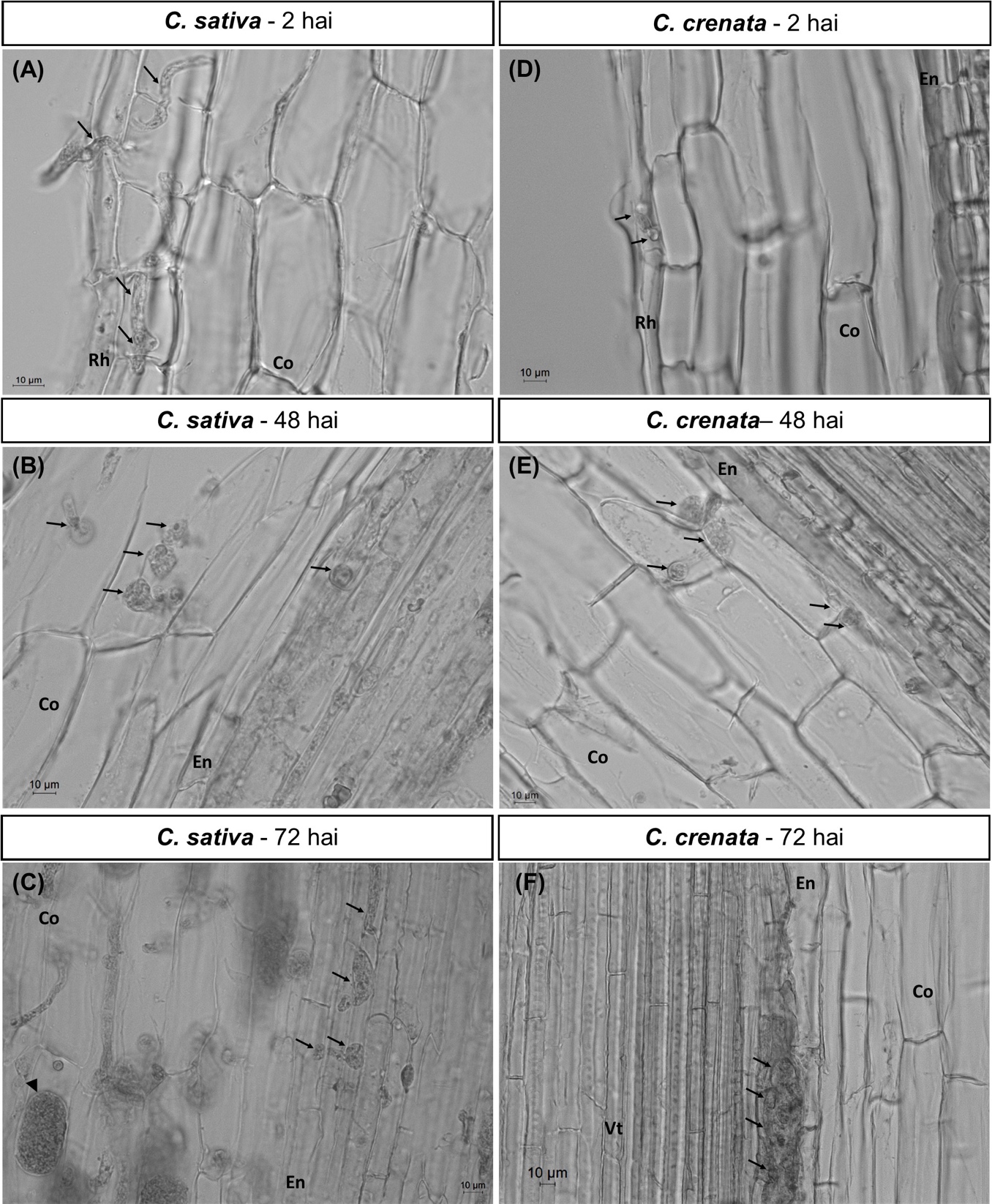


**Supplementary Figure 1.** Light microscopy, cotton blue lactophenol staining. Longitudinal sections of *Castanea sativa* (A, B, C on the left) and *Castanea crenata* (D, E, F on the right) roots after inoculation by direct contact with *Phytophthora cinnamomi* mycelium pieces. (**A**) Hyphae (arrows) penetrating the rhizodermis of *C. sativa* 2h after inoculation (hai). (**B**) Hyphae (arrows) colonizing the cortex cells and the pericycle of *C. sativa* roots 48 hai. (**C**) Hyphae colonizing the pericycle and the adjacent parenchymal cells of the vascular tissues (arrows) of *C. sativa* 72 hai. Some chlamydospores (triangle) were identified in the cortex cells. (**D**) Hypha (arrows) colonizing the rhizodermis of *C. crenata* 2 hai. (**E**) Hyphae (arrows) colonizing the last layers of the cortex cells next to the endodermis in *C. crenata* roots 48 hai. (**F**) Hyphae colonizing the pericycle cells in *C. crenata* roots 72 hai. Bars = 10 µm. Co = cortex, Rh = rhizodermis, En = endodermis, Vt = vascular tissues.


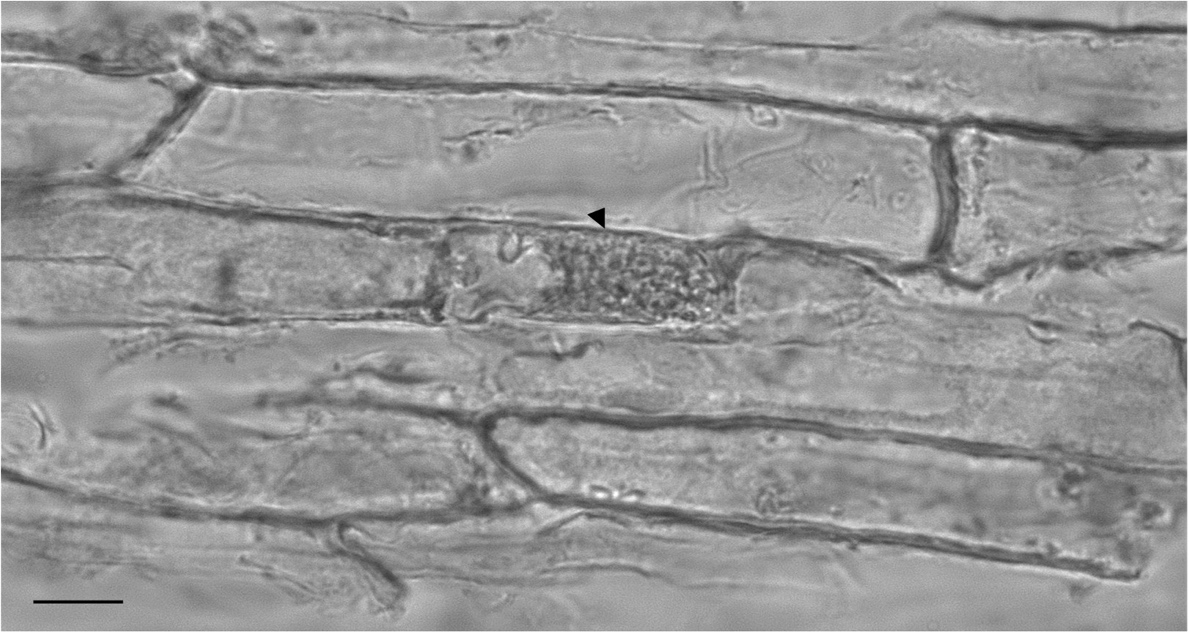


**Supplementary Figure 2.** Light microscopy, cotton blue lactophenol staining. Longitudinal section of *Castanea sativa* roots 72 h after inoculation by direct contact with *Phytophthora cinnamomi* mycelium. Cortical parenchyma site showing cytoplasmic content disorganization. Bar = 10 µm

**
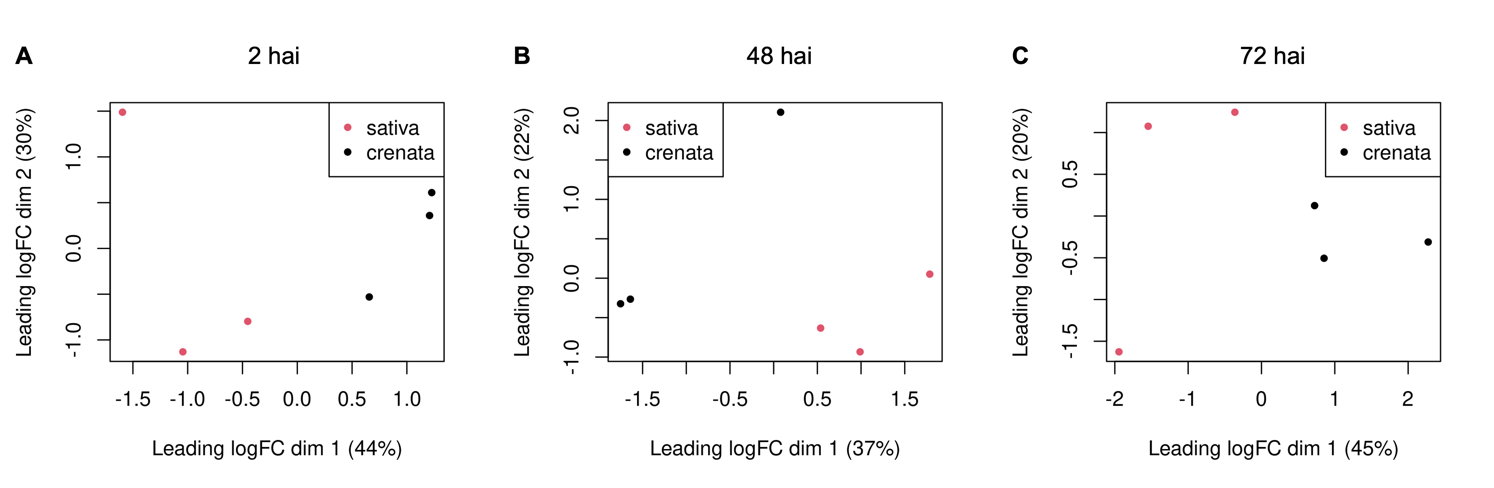
Supplementary Figure 3.** Multidimensional scaling of RNAseq analysis of *Phytophthora cinnamomi* infecting *Castanea crenata* and *Castanea sativa* roots at (**A**) 2, (**B**) 48 and (**C**) 72 hours after inoculation (hai).


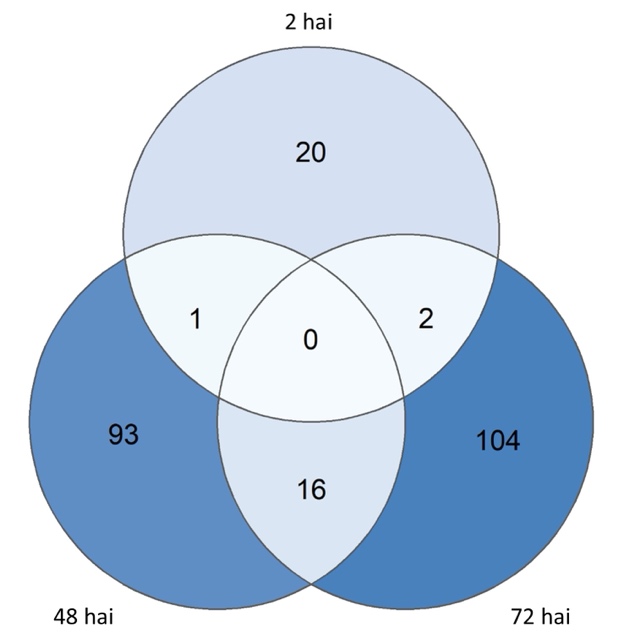


**Supplementary Figure 4.** Venn diagram representing the overlap of differentially expressed genes (DEG) after comparing samples of *Phytophthora cinnamomi* infecting *Castanea crenata* to *Castanea sativa* at 2, 48 and 72 hours after inoculation (hai).


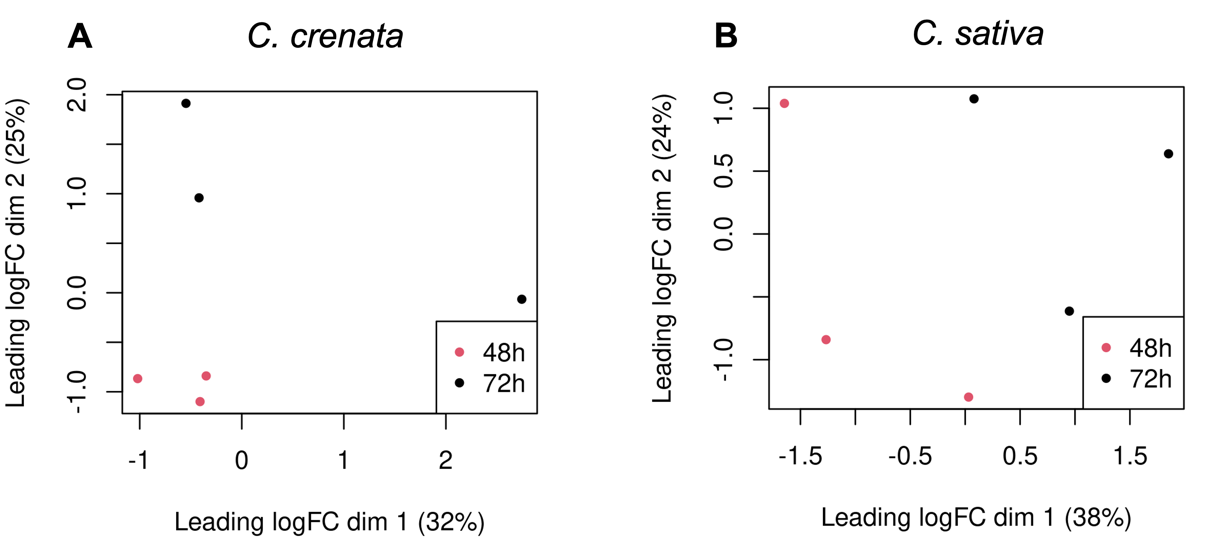


Supplementary Figure 5. Multidimensional scaling of RNAseq analysis of *Phytophthora cinnamomi* infecting *Castanea crenata* (A) and *Castanea sativa* (B) roots at 48 and 72 hours after inoculation.


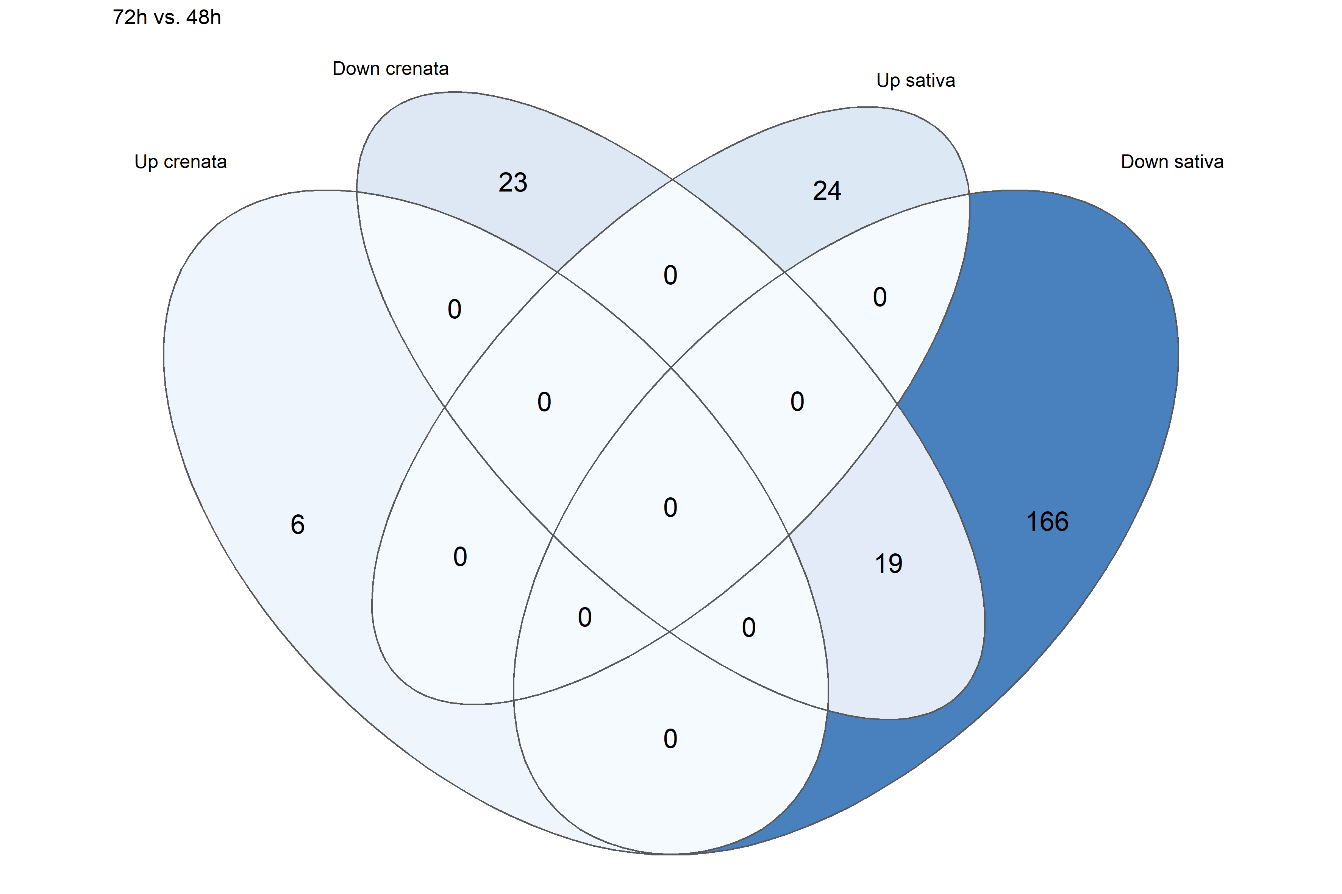


**Supplementary Figure 6.** Venn diagram representing the overlap of differentially expressed genes of *Phytophthora cinnamomi* at 72 hours after inoculation (hai) when compared to 48 hai while infecting *Castanea crenata* or *Castanea sativa*.


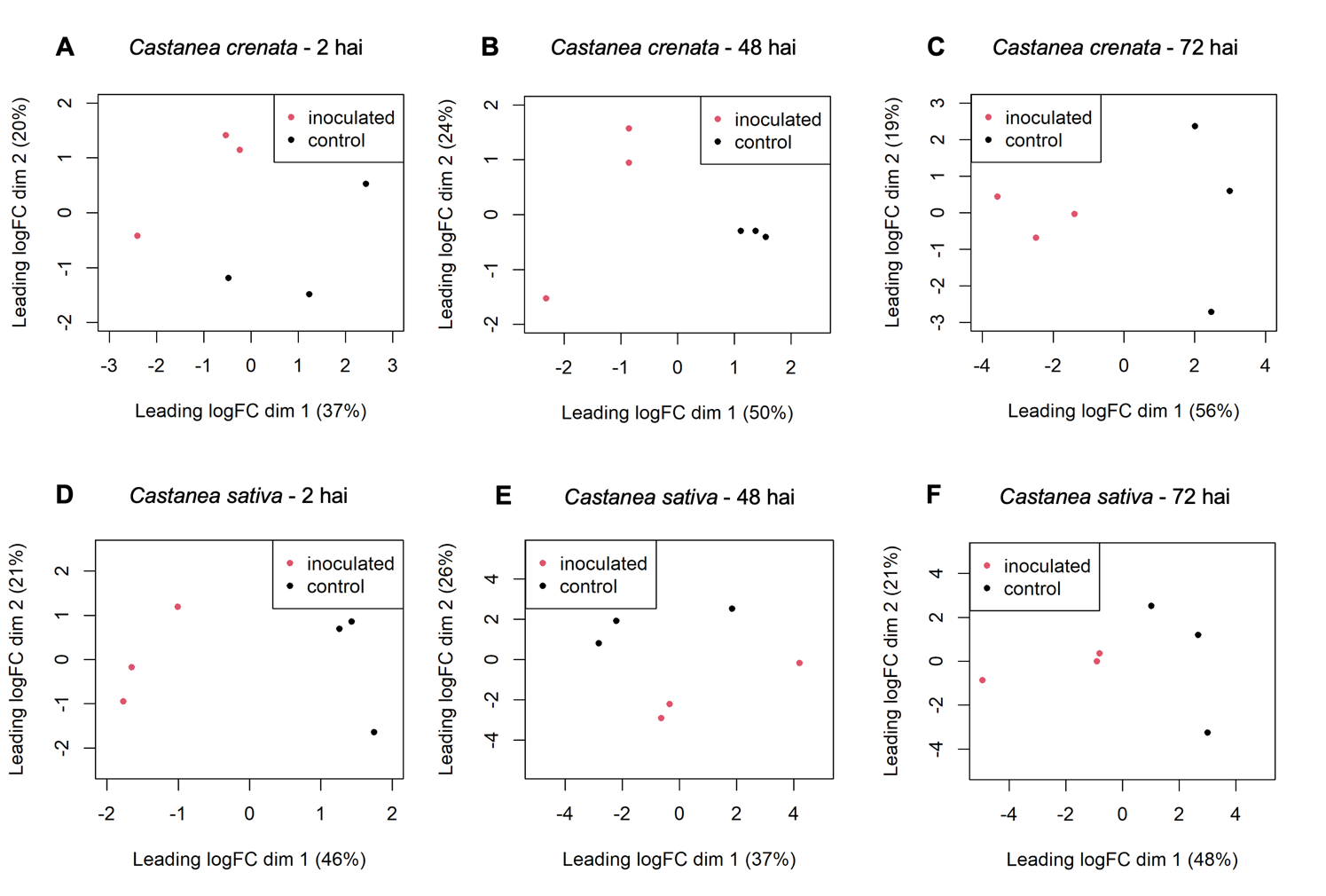
**Supplementary Figure 7.** Multidimensional scaling of RNAseq analysis of *Castanea crenata* and *Castanea sativa* roots infected by *Phytophthora cinnamomi* at (**A**) 2, (**B**) 48 and (**C**) 72 hours after inoculation (hai).


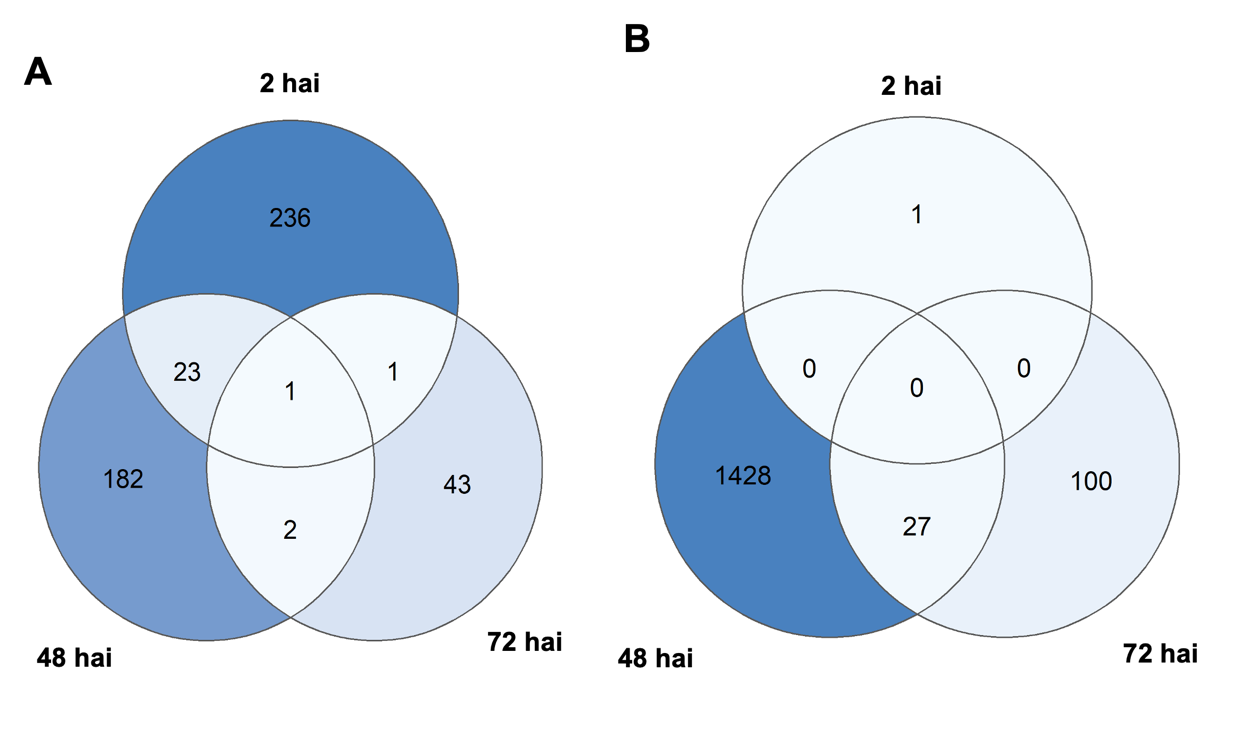


**Supplementary Figure 8.** Venn diagrams showing overlapping differentially expressed genes of *C. sativa* (**A**) and *C. crenata* (**B**) after comparing *P. cinnamomi* inoculated samples with non-inoculated controls.
